# Critical role for the TGF-β1/mTORC1 signaling axis in defining the transcriptional identity of CTHRC1^+^ pathologic fibroblasts

**DOI:** 10.1126/sciadv.adx9868

**Published:** 2026-09-02

**Authors:** Jo-Anne AM Wilson, Rachel Walters, Greg Contento, Madeleine Rossanese, Delphine Guillotin, Alexander I. Ward, Robert E. Hynds, Mariam Jamal-Hanjani, Naftali Kaminski, Silvia Parolo, Manuela Platé, Rachel C. Chambers

**Affiliations:** ^1^ Centre for Inflammation and Tissue Repair, UCL Respiratory, University College London, London WC1E 6JF, UK.; ^2^ Epithelial Cell Biology in ENT Research (EpiCENTR) Group, Developmental Biology and Cancer Department, Great Ormond Street UCL Institute of Child Health, University College London, London, UK.; ^3^ Cancer Research UK Lung Cancer Centre of Excellence, University College London Cancer Institute, London, UK.; ^4^ Cancer Metastasis Laboratory, University College London Cancer Institute, London, UK.; ^5^ Department of Medical Oncology, University College London Hospitals, London, UK.; ^6^ Section of Pulmonary, Critical Care and Sleep Medicine, Yale School of Medicine, New Haven, CT, USA.; ^7^ Fondazione The Microsoft Research - University of Trento Centre for Computational and Systems Biology (COSBI), Rovereto, Italy.

## Abstract

Fibrosis, marked by excess extracellular matrix (ECM) deposition, is the end stage of many diseases. Single-cell studies have highlighted the emergence of disease-specific fibroblast populations, including a high collagen–synthesizing *CTHRC1^+^* subpopulation. The profibrotic cytokine TGF-β1 promotes fibrogenesis via cooperation between Smad and mTORC1/4E-BP1 signaling axes. Using CRISPR-Cas9 gene editing, we report that more than one-third of TGF-β1–regulated matrisome genes are under mTORC1 control. Mapping the transcriptome of TGF-β1–stimulated fibroblasts revealed similarity to *CTHRC1^+^* fibroblasts identified in idiopathic pulmonary fibrosis (IPF). This overlap is lost when mTORC1 is disabled. Using the selective mTORC1 inhibitor RMC-5552, we confirm a causal role for mTORC1 in promoting the acquisition of the collagen-high, *CTHRC1^+^* phenotype in response to TGF-β1 stimulation in fibroblasts derived from patients with either IPF or lung adenocarcinoma. We conclude that mTORC1 plays a key role in shaping the transcriptional identity of these fibroblasts, with implications for therapeutic inhibition of mTORC1 in fibrosis and cancer.

## INTRODUCTION

Fibrosis is a highly conserved tissue injury response and represents the concluding pathological outcome in several common chronic inflammatory, immune-mediated, and metabolic diseases. Pathological fibrosis can affect any organ and accounts for up to 45% of deaths annually in the industrialized world (*1*). In terms of progression to organ failure, idiopathic pulmonary fibrosis (IPF) represents one the most rapidly progressive and fatal fibrotic conditions with a life expectancy of 3 to 5 years from diagnosis despite the introduction of the first approved antifibrotic agents, pirfenidone and nintedanib, which slow but do not halt disease progression (*2*–*4*). Fibrotic foci (also known as fibroblastic foci) represent the cardinal pathogenic lesions in IPF and comprise activated fibroblasts, the key effector cells responsible for dysregulated extracellular matrix deposition. While the etiology of IPF remains elusive, current evidence suggests that this condition arises as a consequence of a combination of genetic risk factors and environmental exposures and manifests in aged individuals as a highly abnormal wound healing response characterized by dysregulated epithelial-mesenchymal cross-talk (*5*). Recent single-cell RNA sequencing (scRNA-Seq) studies have uncovered the diversity and functional complexity of fibroblast subtypes and have identified multiple fibroblast subpopulations which are only present in fibrosis (*6*, *7*). Notably, this includes a recently identified population of high collagen–producing pathological fibroblasts, characterized by high expression of the secreted glycoprotein, collagen triple helix repeat containing 1 (CTHRC1), which plays a pivotal role in extracellular matrix (ECM) deposition and wound repair (*8*–*10*), hereafter collagen-high *CTHRC1^+^* fibroblasts.

Although the triggers of the aberrant fibrogenic response vary between tissues, there are core mechanisms shared across multiple organs (*11*–*13*). This includes persistent signaling by transforming growth factor β1 (TGF-β1), the most potent fibrogenic cytokine characterized to date. TGF-β1 controls the activation of fibroblasts and promotes ECM deposition by up-regulating the expression of an extensive profibrotic transcriptional program involving multiple matrisome and matrisome-associated genes (*14*). More recently, CTHRC1 has been reported to be a primary target for TGF-β1 signaling in the fibrotic microenvironment in several fibrotic conditions (*15*).

TGF-β1 signals through the canonical Smad pathway and several noncanonical signaling pathways, and we have previously reported that the canonical Smad pathway cooperates with mTOR signaling to mediate the potent fibrogenic effects of TGF-β1 in primary lung fibroblasts derived from control subjects and patients with IPF (*16*). Targeted knockdown of Smad3 in primary human lung fibroblasts (pHLFs) attenuates mTOR complex 1 (mTORC1)–dependent phosphorylation of its downstream substrates p70S6K and 4E-BP1, indicating that the activation of mTORC1 is dependent on Smad3, whereas, the phosphorylation of either Smad2 or 3 is not dependent on mTOR signaling (*16*). The observation regarding the key role of mTORC1 during fibrogenesis was extended to other cell types, including hepatic stellate cells and skin fibroblasts, as well as cancer-associated fibroblasts (CAFs) derived from lung adenocarcinoma, as part of ongoing efforts to identify therapeutic strategies to disrupt the stromal reaction in cancer (*16*).

mTOR is a nodal serine/threonine kinase that is present in two similarly organized but functionally distinct signaling complexes, mTORC1 and mTORC2. The two mTOR complexes share several accessory proteins (Deptor, MLST8, Tti1, and Tel2), with mTORC1 also containing Raptor and PRAS40 and mTORC2 containing Rictor, Protor1/2 and mSin1. The accessory subunits endow each complex with distinct modes of substrate recognition and regulation, which in turn dictates their downstream cellular activities. mTORC1 integrates signals such as growth factors and the availability of nutrients, energy, and oxygen and triggers cellular responses including protein translation, lipid biosynthesis, senescence, and autophagy to either boost anabolism or suppress catabolism via the phosphorylation of ribosomal protein S6 kinase beta-1 (S6K1), also known as p70S6 kinase (p70S6K) and eukaryotic translation initiation factor 4E–binding protein 1 (4E-BP1), among others. mTORC2 is responsive to growth factor signaling, and its functions include the regulation of cell proliferation, survival, and cytoskeletal remodeling via the activation of protein kinase B (AKT), serum and glucocorticoid-induced protein kinase 1 (SGK1), and protein kinase C (PKC). Our previous exploration of the role of mTOR signaling during TGF-β1–induced collagen synthesis, revealed a key role for the mTORC1/4E-BP1 axis (*16*), so that therapeutic targeting of this axis may offer an antifibrotic treatment approach.

The mTOR axis has been implicated in multiple cancers, as well as diabetes and neurodegenerative disease, and this axis has been avidly pursued as a therapeutic target in these disease settings. First generation allosteric inhibitors, such as rapamycin and its analogs (rapalogs), bind to the mTOR FKBP-rapamycin binding domain (FRB) and are partial inhibitors in that they prevent the phosphorylation activity of mTORC1 with preferential impact on the phosphorylation of its major downstream substrate, p70S6K. Because of its active site being partially blocked by Rictor, mTORC2 is unaffected unless exposure is prolonged (*17*). Since the mTORC1-dependent effects of TGF-β1 on collagen synthesis are 4E-BP1–mediated, this response is unaffected by rapamycin treatment (*16*). Second and third generation mTOR inhibitors comprise dual mTORC1 and mTORC2 adenosine 5′-triphosphate (ATP)–competitive mTOR inhibitors. These function by blocking the mTOR ATP-binding pocket and are currently being explored for their potential in treating various cancers (*18*). More recently, the generation of first-in-class class selective bisteric inhibitors of mTORC1, which interact with both the orthosteric and the allosteric binding sites to deepen the inhibition of mTORC1 while also preserving selectivity for mTORC1 over mTORC2, have been developed. These mTORC1 selective inhibitors potently inhibit p70S6K and 4E-BP1 phosphorylation while limiting unwanted effects on glucose metabolism and providing relief of AKT-dependent feedback inhibition of receptor tyrosine kinase (RTK) expression as a result of concomitant mTORC2 inhibition (*19*). At the time of writing, the lead compound, RMC-5552, is a clinical-phase asset in the oncology setting (*20*). The aim of the current study was to investigate the role of the mTOR signaling hub in regulating the global transcriptional response to TGF-β1 and further to determine whether mTOR contributes to the transcriptional identity of a high collagen expressing *CTHRC1*^+^ fibroblast population.

## RESULTS

### ATP-competitive inhibition of mTOR attenuates TGF-β1–induced extracellular matrix gene expression

We have recently provided strong support for mTORC1 as a key signaling node in regulating TGF-β1–induced collagen I production (*16*). Recent scRNA-Seq studies have identified several pathological fibroblast subpopulations, which are present in multiple fibrotic conditions, including IPF (*6*, *7*). However, whether mTORC1 acts as a master transcriptional regulator of the ECM and plays a role in shaping the functional complexity of fibroblast subpopulations, including the acquisition of the *CTHRC1^+^* fibroblast phenotype in response to TGF-β1 signaling in the fibrotic niche, is not known. To gain further insight into these questions, we first examined an existing RNA-Seq dataset from our laboratory (GSE102674) (*21*), conducting differential gene expression analysis to compare the effect of dual mTOR inhibition using the ATP-competitive mTOR inhibitor, AZD8055, with the partial mTORC1 allosteric inhibitor, rapamycin, on the TGF-β1–induced transcriptome in pHLFs.

**Fig. 1.**
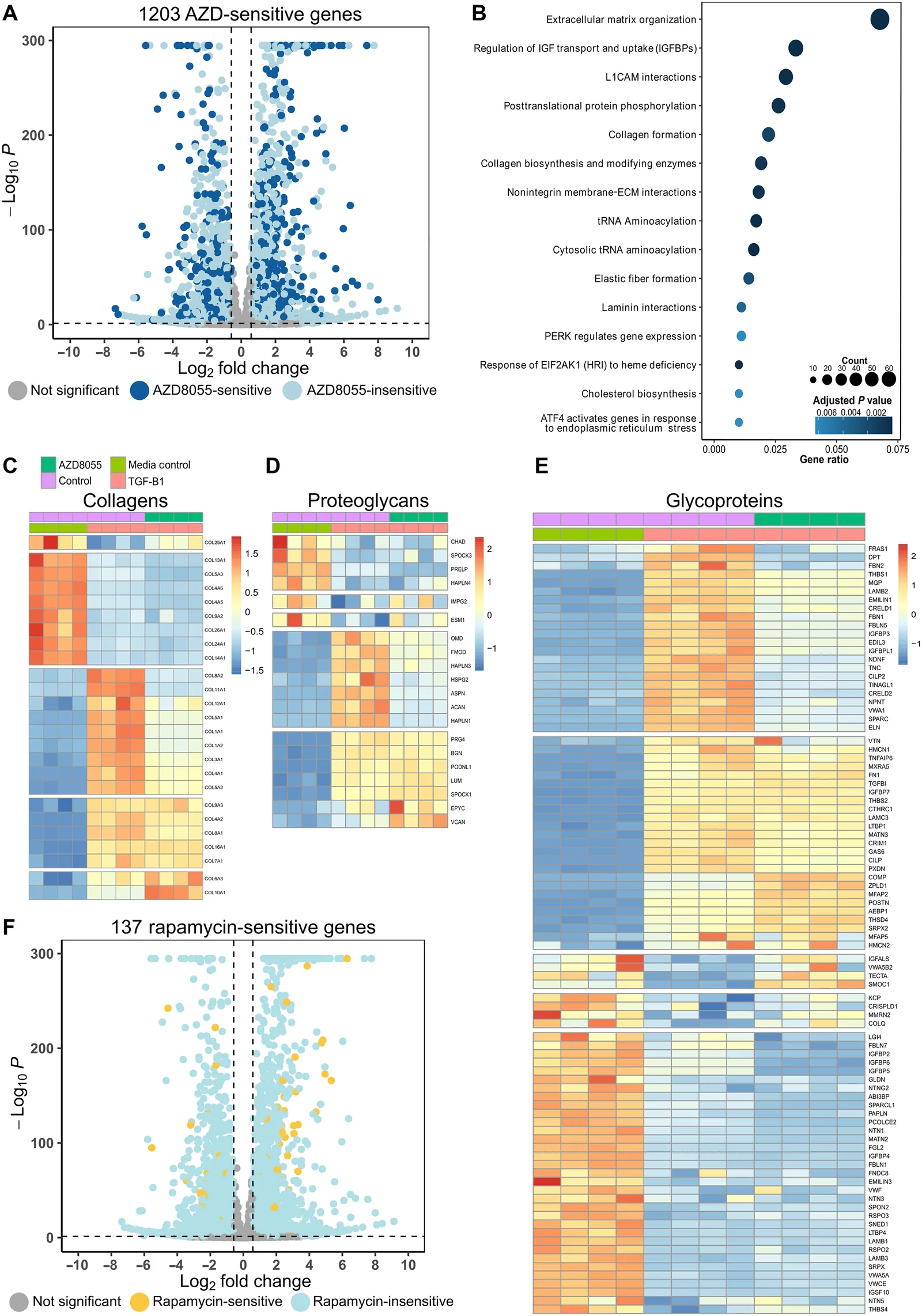
ATP-competitive mTOR inhibition attenuates the TGF-β1–induced transcriptional response in primary human lung fibroblasts. pHLFs were incubated with vehicle [0.1% dimethyl sulfoxide (v/v)], AZD8055 (1 μM), or rapamycin (100 nM) for 1 hour before stimulation with or without TGF-β1 (1 ng/ml) for 24 hours, and RNA-seq was performed (GSE102674) (*21*) followed by standard interaction analysis. (**A**) Volcano plot depicting the TGF-β1 response, with 1203 AZD8055-sensitive genes shown in dark blue and AZD8055-insensitive genes shown in light blue (adjusted *P* value < 0.05 and fold change > 1.5). (**B**) Dot plot showing the top 15 Reactome pathways (adjusted *P* value < 0.01) from overrepresentation analysis of the AZD8055/TGF-β1–dependent genes. Count refers to the number of genes in the dataset that are part of that pathway, and gene ratio refers to the ratio of the number of genes within that pathway that are differentially expressed to the total number of genes in that pathway. (**C** to **E**) Heatmaps representing supervised clustering analysis of collagen, proteoglycan, and glycoprotein genes of vehicle-treated control, TGF-β1–stimulated, and TGF-β1/AZD8055–treated pHLFs. Color scale represents normalized counts scaled to *z*-score. (**F**) Volcano plot depicting the TGF-β1 response, with 137 rapamycin-sensitive genes in yellow and rapamycin-insensitive genes in light blue (adjusted *P* value < 0.05 and fold change > 1.5). Figure shows data from a single experiment with *n* = 4 technical replicates. For volcano plots [(A) and (F)], genes appearing at the top of the volcano plots have *P* values below the computational precision limit, corresponding to maximal statistical significance. tRNA, transfer RNA.

The differential gene expression analysis of pHLFs identified 4911 genes that were significantly modulated in response to TGF-β1, of which 2258 genes were significantly (adjusted *P* value <0.05) up-regulated with fold change > 1.5 and 2653 genes were significantly down-regulated with fold change < −1.5. These genes are listed in the interactive table S1 to enable readers to run their own future queries on our datasets. Within the TGF-β1 responsive genes, we identified 1203 genes that exhibited sensitivity to AZD8055 through standard interaction analysis (Fig. 1A), indicating that up to 24.5% of genes affected by TGF-β1 are likely under mTOR regulatory control (for a full list please see interactive table S2). The pathways analysis of these differentially expressed genes (DEGs) revealed that the most overrepresented AZD8055-sensitive pathways were critically linked to the fibrotic response (Fig. 1B). The top pathway, extracellular matrix organization, encompassed more than 60 ECM and ECM-related genes. In addition, pathways associated with collagen formation and biosynthesis, elastic fiber formation, and ECM interactions ranked among the top 15. The pathway “ATF4 activates genes in response to endoplasmic reticulum stress” was also significantly enriched, aligning with previous findings from our group showing a critical role for the mTOR-dependent ATF4 pathway during fibrogenesis (*21*). Furthermore, critical ECM genes including collagens, proteoglycans, and glycoproteins were up-regulated by TGF-β1 and counter-modulated by AZD8055 treatment (Fig. 1, C to E).

The exploration of the effect of the partial mTORC1 allosteric inhibitor, rapamycin, on the TGF-β1–induced ECM in the same dataset (GSE102674) revealed that only 137 TGF-β1–responsive genes were found to be sensitive to rapamycin. This represents just 2.5% of the total TGF-β1–responsive genes (Fig. 1F; for a full gene list, please see interactive Supplementary Tables). Pathways analysis of these rapamycin-sensitive DEGs revealed that they are overrepresented in two pathways: regulation of cholesterol biosynthesis by SREBP (SREBF) and metabolism of steroids (fig. S1A). Only a very small number of rapamycin-sensitive matrisome genes, with the majority being secreted factors, were identified (fig. S1B).

### The TGF-β1–induced profibrotic gene program is under mTORC1 regulatory control

The large number of AZD8055-sensitive genes identified within the TGF-β1–induced transcriptome led us to next determine the relative contributions of the two individual mTOR complexes to this response. To this end, we subjected pHLFs to CRISPR-Cas9 gene editing of either *RPTOR* or *RICTOR* to disable mTORC1 and mTORC2 signaling, respectively, and performed RNA-Seq in cells exposed to TGF-β1 for 24 hours. The successful reduction in *RPTOR* and *RICTOR* expression by CRISPR-Cas9 gene editing and disruption of downstream signaling in response to TGF-β1 stimulation is shown in fig. S2 (A and B, respectively). Also shown is the responsiveness of gene-edited cells in terms of ability to mount a TGF-β1–induced collagen response (fig. S2C), supporting our previous observation that *RPTOR* is essential for TGF-β1–induced collagen synthesis, whereas *RICTOR* is dispensable.

**Fig. 2.**
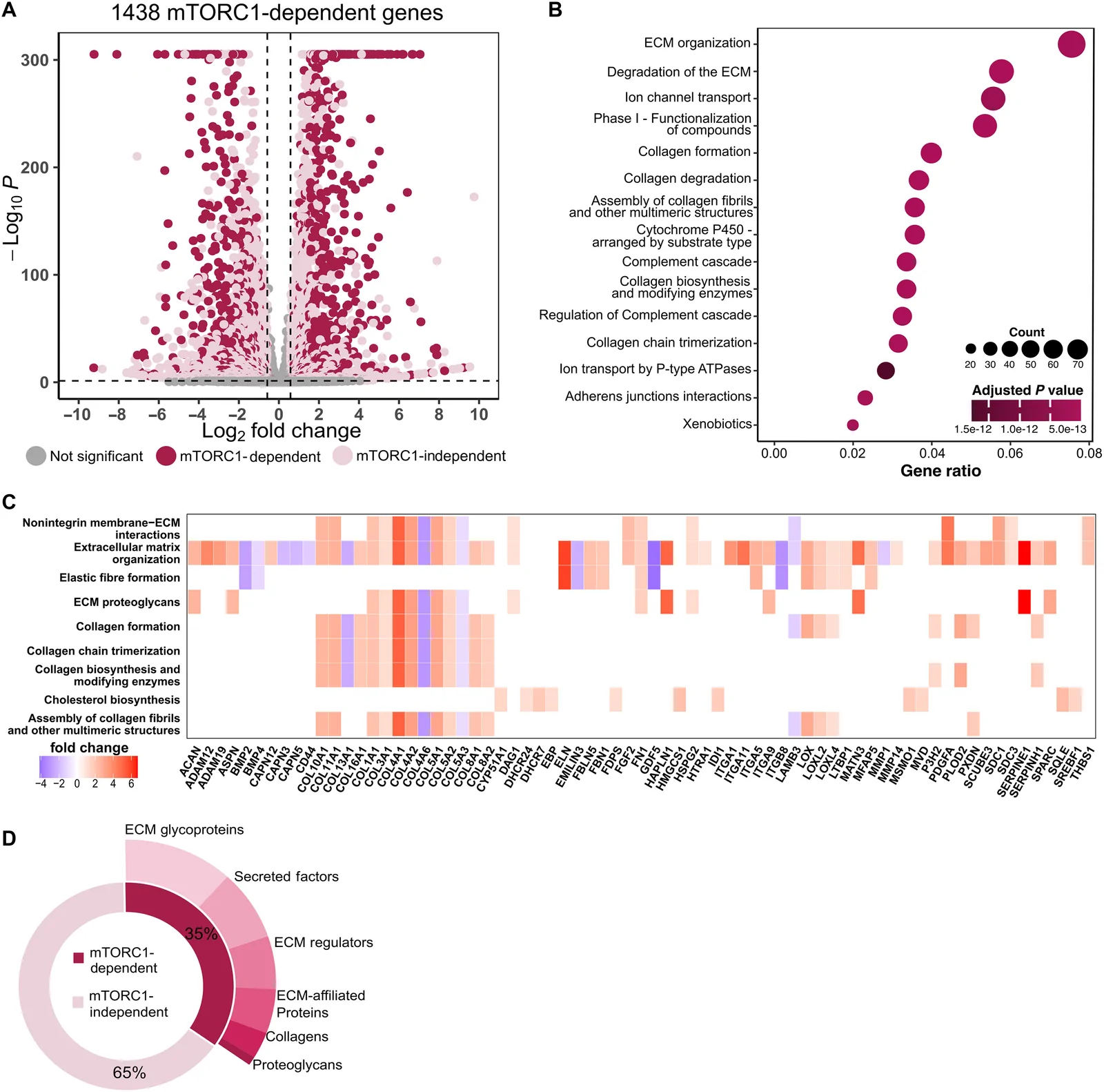
RNA-Seq analysis in combination with CRISPR-Cas9 gene editing reveals that the TGF-β1–induced fibrotic response is under mTORC1 regulatory control. pHLFs were modified using CRISPR-Cas9 gene editing with guide RNAs targeting *RPTOR* or *RICTOR.* Cells were then stimulated with or without TGF-β1 (1 ng/ml) for 24 hours and RNA-Seq was carried out followed by standard interaction analysis. (**A**) Volcano plot showing DEGs following TGF-β1 stimulation (adjusted *P* value < 0.05 and absolute fold change > 1.5) with 1438 mTORC1-dependent genes highlighted in maroon and mTORC1-independent genes in light pink. Genes appearing at the top of the volcano plots represent *P* values below the computational precision limit, corresponding to maximal statistical significance. (**B**) Dot plot showing the top 15 Reactome pathways (adjusted *P* value < 0.01) from overrepresentation analysis on the TGF-β1/mTORC1-dependent genes. Dot size represents the number of enriched genes, and the *x* axis represents the fraction of enriched genes over the total number in the Reactome gene set. The color scale represents the adjusted *P* value. (**C**) Heat plot showing gene membership of the mTORC1-dependent ECM pathways from the top 15 presented in (B). The color scale represents the TGF-β1–induced fold change of a given gene within a pathway. (**D**) Doughnut chart showing the number of TGF-β1–dependent matrisomal genes that are mTORC1-dependent (counter-modulated in CRISPR-Cas9 *RPTOR* gene-edited cells with adjusted *P* value < 0.05 and absolute fold change > 1.5). Figure represents data from a single experiment with *n* = 3 technical replicates. ATPases, adenosine triphosphatases.

Differential gene expression analysis revealed that of the genes regulated at the transcriptional level by TGF-β1, only 13 genes were mTORC2 dependent (for a list of these genes please see interactive, Supplementary Tables), and there were no significant mTORC2-dependent pathways identified through pathways analysis. In contrast, 1438 of the TGF-β1–responsive genes (Fig. 1A) were mTORC1 dependent (Fig. 2A; for a full gene list, please see interactive Supplementary Tables). The top overrepresented 15 mTORC1-dependent pathways comprise those critical to fibrogenesis (Fig. 2B), including the top pathway of extracellular matrix organization with 60 associated genes. The DEGs mapping to these ECM-related pathways are depicted in Fig. 2C. When classifying these genes as either matrisome or nonmatrisome genes, we found that of the TGF-β1–dependent matrisome genes, 35% were under mTORC1-dependent transcriptional control (Fig. 2D). This includes genes encoding the pro-alpha 1 chain of the two most prevalent collagen types within the ECM, *COL1A1* and *COL3A1*, and other fibrillar collagens (e.g., *COL5A1*, *COL5A2*, and *COL11A1*), as well as several collagen cross-linking genes (e.g., *PLOD2*, *LOX*, and *LOXL2*). Furthermore, genes that are associated with the mTOR axis were also TGF-β1 and mTORC1 regulated (e.g. *EIF4EBP1*, *EIF4B*, and *RHEB*), suggesting a potential feedback loop in the regulation of the mTOR signaling hub. We also applied the QIAGEN IPA Upstream Regulatory Analysis (URA) tool to the list of TGF-β1/mTORC1-dependent genes to identify potential transcriptional regulators that had been previously reported, through experimental evidence, to affect the expression of our set of genes. The results of this analysis are presented in table S3, with the top 30 predicted transcriptional regulators shown. This includes several transcription factors already associated with TGF-β1 signaling and ECM production (*14*, *21*–*23*).

In contrast, the top TGF-β1–regulated but mTORC1-independent DEGs (total of 5684 genes) map to nine significant Reactome pathways (fig. S3). Of these, the Rho guanosine triphosphatase cycle is noteworthy as this pathway has been linked to stress fiber formation and myofibroblast contractility in the setting of fibrosis, so that while mTORC1 plays a key role in the regulation of ECM biosynthesis, genes encoding RhoA and RhoD involved in controlling fibroblast cytoskeletal contraction following TGF-β1 stimulation are mTORC1 independent.

### The TGF-β1/mTORC1 axis plays a critical role in defining the transcriptional identity of a high collagen–producing *CTHRC1*^+^ fibroblast subpopulation in IPF

**Fig. 3.**
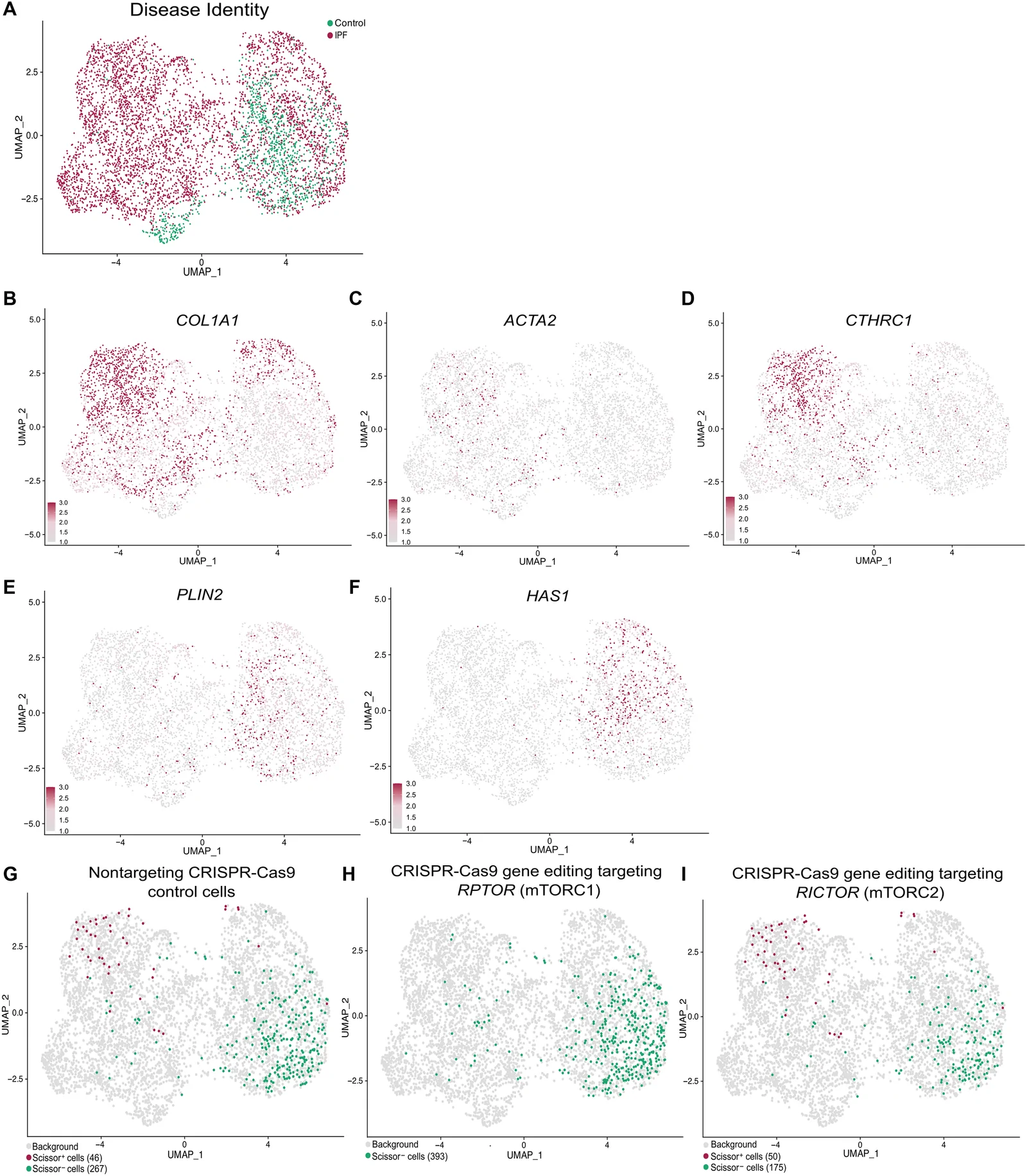
Integration of IPF scRNA-seq and in vitro pHLF RNA-seq data reveals a role for the TGF-β1/mTORC1 axis in defining the transcriptional identity of the *CTHRC1*^+^ fibroblast population present in the IPF lung. Scissor was used to map RNA-Seq data (from Fig. 2) onto a human IPF scRNA-Seq dataset (GSE136831) (*24*). (**A**) UMAP visualization of the fibroblasts from the scRNA-Seq dataset, colored according to disease status. Control is shown in green and IPF in maroon. (**B** to **F**) UMAP visualization of the expression levels of key activated fibroblast markers reported in the literature, including *COL1A1* (B), *ACTA2* (C), *CTHRC1* (D), *PLIN2* (E), and *HAS1* (F). (**G** to **I**) UMAP visualization of the Scissor-selected cells corresponding to nontargeting CRISPR-Cas9 control cells (G), CRISPR-Cas9 *RPTOR* gene-edited cells (H), and CRISPR-Cas9 *RICTOR* gene-edited cells (I), where the maroon and green dots are cells associated with TGF-β1–stimulated (Scissor^+^) and control-media (Scissor^−^) cells, respectively, while background cells are gray.

Following the observation that a large number of profibrotic genes in TGF-β1–stimulated cells are regulated by mTORC1, we next explored whether mTOR might play a role in shaping the functional complexity of fibroblast subpopulations in the fibrotic niche. To this end, we integrated our RNA-Seq dataset obtained from TGF-β1–stimulated *RPTOR* and *RICTOR* gene-edited pHLFs (Fig. 2) with existing, publicly available human IPF scRNA-Seq datasets. In addition to the more traditional markers of activated fibroblasts, *COL1A1* and *ACTA2* (*7*, *8*), more recently described markers of high collagen–expressing fibroblast populations in IPF include *CTHRC1* and *HAS1*. Fibroblasts and myofibroblasts were extracted from the human IPF scRNA-Seq dataset GSE136831, with disease status (IPF = maroon versus control = green) for each cell shown in Fig. 3A. Figure 3 (B to F) shows the expression of each of the above markers in the reference human scRNA-Seq dataset (GSE136831) (*24*). This analysis is consistent with previous reports that identified *CTHRC1* as a more specific marker of the fibroblast population that produces the highest levels of ECM proteins in pulmonary fibrosis and other fibrotic diseases (*8*, *25*).

We then applied the bioinformatics tool Scissor (*26*), which is a recently developed approach that uses the phenotypes collected from bulk assays to identify the most highly phenotype-associated cell subpopulations from single-cell data. We first applied this tool to allow the identification of the IPF fibroblast subpopulation with a transcriptome that best matches the TGF-β1–induced transcriptome of pHLFs in nongene-edited control cells (i.e., nontargeting CRISPR-Cas9 control group treated with TGF-β1). We then explored this same question in pHLFs in which either mTORC1 (CRISPR-Cas9 *RPTOR* gene-edited group) or mTORC2 signaling (CRISPR-Cas9 *RICTOR* gene-edited group) is disabled. Scissor analysis revealed that the baseline transcriptome of in vitro cultured pHLFs closely matches that of freshly isolated fibroblasts from human lungs characterized by low expression of *CTHRC1* and *COL1A1* (Scissor^−^ cells from the nontargeting CRISPR-Cas9 control group; Fig. 3G). In contrast, the transcriptome of TGF-β1–stimulated pHLFs overlaps with cells within a population of fibroblasts unique to IPF, characterized by the high expression of *CTHRC1* and *COL1A1* (Scissor^+^ cells from the nontargeting CRISPR-Cas9 control group; Fig. 3G). The analysis of the distribution of Scissor^+^ and Scissor^−^ cells according to disease status further revealed that 100% of the Scissor^+^ fibroblasts map to IPF fibroblasts from 15 different human IPF lungs, with the majority of Scissor^+^ fibroblasts representing myofibroblasts (table S1). For the Scissor^−^ cells, the split is less marked in terms of disease status; with 54% mapping to IPF and 46% mapping to non-IPF control fibroblasts. Strikingly, the transcriptome of TGF-β1–stimulated pHLFs in which mTORC1 signaling has been disabled (Scissor^+^ cells from the CRISPR-Cas9 *RPTOR* gene-edited group; Fig. 3H), no longer maps to the *CTHRC1*^+^ fibroblast population or any other fibroblast population present in human lungs captured through scRNA-Seq (Scissor^+^ cells from the CRISPR-Cas9 *RPTOR* gene-edited group; Fig. 3H). In contrast, similar to the nontargeting CRISPR-Cas9 control group (Scissor^−^ cells from the nontargeting CRISPR-Cas9 control group; Fig. 3G), the transcriptional profile of baseline *RICTOR* gene-edited pHLFs (Scissor^−^ cells from the CRISPR-Cas9 *RICTOR* gene-edited group; Fig. 3I) closely matches that of freshly isolated fibroblasts from human lungs characterized by low expression of *CTHRC1* and *COL1A1*. Moreover, the TGF-β1–stimulated *RICTOR* CRISPR-Cas9 group (Scissor^+^ cells from the CRISPR-Cas9 *RICTOR* gene-edited group, Fig. 3I) in turn maps to the high *CTHRC1*^+^ group, suggesting that mTORC2 is dispensable for the acquisition of the pathological *CTHRC1*^+^ fibroblast phenotype. We also extended the Scissor analysis to an additional human IPF scRNA-Seq dataset (GSE135893) (*7*). This analysis confirmed the observation that mTORC1 signaling is necessary for defining the transcriptional identity of the *CTHRC1*^+^ pathological fibroblast population in response to TGF-β1 signaling in vitro and in the fibrotic niche in IPF (fig. S5).

Our next aim was to characterize the identity of the Scissor^−^ cells mapping to our in vitro fibroblast population at baseline. We therefore carried out differential gene expression analysis of the Scissor^−^ cells from the nontargeting CRISPR-Cas9 control group versus all other fibroblasts in human lung. We identified 159 genes significantly (adjusted *P* value < 0.05) up-regulated or down-regulated (absolute fold change > 1.5), which are reported in interactive Supplementary Tables. Based on this analysis, the pHLFs at baseline (derived by explant culture of control lung parenchyma) likely represent a low ECM expressing phenotype and expression of markers associated with both adventitial and alveolar fibroblasts. Alveolar identity is supported by expression of *MFAP5* (microfibrillar-associated protein 5) and *SCARA5* (scavenger receptor class A member 5), which together define mesenchymal alveolar niche cells that provide critical support for type II alveolar epithelial cells (*27*). Adventitial fibroblast identity is marked by *PI16* (peptidase inhibitor 16) (*28*), a defining feature of perivascular adventitial fibroblasts, together with *CD34* (cluster of differentiation 34) (*28*), and *SFRP1/2/4* (secreted frizzled-related proteins 1, 2, and 4), which modulate Wnt signaling in perivascular niches (*29*, *30*).

**Fig. 4.**
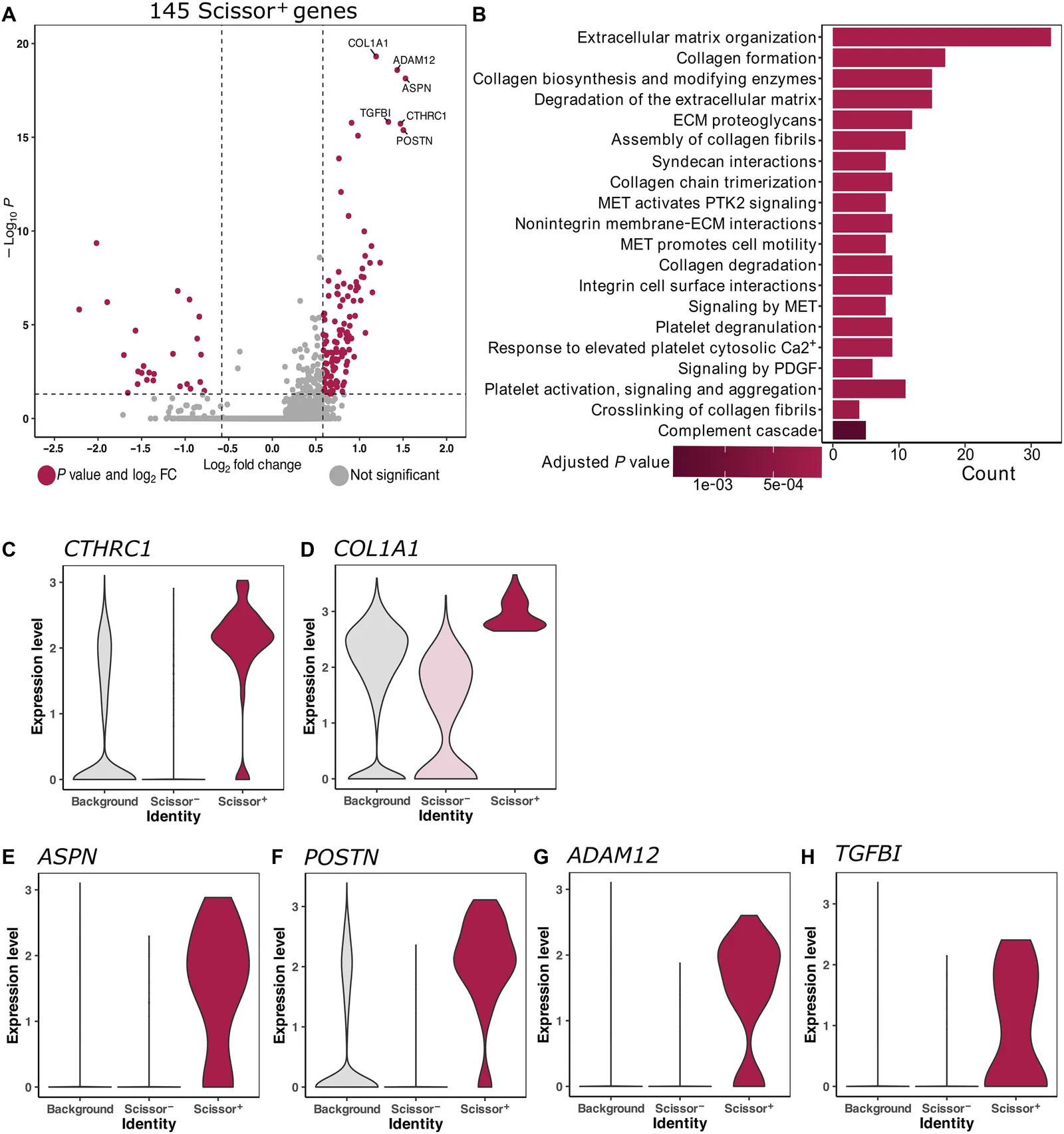
Key pathways and genes defining the transcriptional identity of the *CTHRC1*^+^ fibroblast population are dependent on mTORC1 signaling. Scissor was used to map RNA-Seq data (from Fig. 2) onto a human IPF scRNA-Seq dataset (GSE136831). (**A**) Volcano plot highlights the DEGs for the Scissor^+^ population, when compared to all other fibroblasts. Significant genes are highlighted in maroon (adjusted *P* value < 0.05 and absolute fold change > 1.5). (**B**) Bar plot showing the top 20 Reactome pathways (adjusted *P* value < 0.01) from overrepresentation analysis of the Scissor^+^ genes. The *x* axis represents the number of genes enriched in that pathway, and the color scale represents the adjusted *P* value. (**C** to **H**) Violin plots show the expression of the six genes: (C) *CTHRC1*, (D) *COL1A1*, (E) *ASPN*, (F) *POSTN*, (G) *ADAM12*, and (H) *TGFBI*, in the Scissor^+^ population (maroon) with the highest fold changes in comparison to the Scissor^−^ (light pink) and all other fibroblasts in the dataset (gray). The six genes are significantly up-regulated upon TGF-β1 stimulation in the differential gene expression analysis of the RNA-Seq dataset and counter-modulated in CRISPR-Cas9 *RPTOR* gene-edited cells (adjusted *P* value < 0.05 and absolute fold change > 1.5). PDGF, platelet-derived growth factor.

We then focused on identifying the key pathways and genes that define the transcriptional identity of the TGF-β1–regulated *CTHRC1*^+^ pathological fibroblast population. Differential gene expression analysis was applied to the IPF scRNA-seq data (from GSE136831) comparing the Scissor^+^ cells from the nontargeting CRISPR-Cas9 control group versus all other fibroblasts in human lung (Fig. 3G). We identified 145 genes significantly (adjusted *P* value < 0.05) up-regulated or down-regulated (absolute fold change > 1.5), as shown in Fig. 4A (for a full gene list please, see the interactive Supplementary Tables). The pathways analysis of the 145 DEGs identified for the Scissor^+^ population revealed that the pathways most associated with the Scissor^+^ population (nontargeting CRISPR-Cas9 control group) versus all other fibroblasts in human lung were predominantly related to extracellular matrix organization and collagen synthesis (Fig. 4B). We identified six genes with the highest positive fold changes that define the transcriptional identity of the *CTHRC1*^+^ fibroblast population, including *CTHRC1* and *COL1A1*, as expected (Fig. 4, C and D), as well as *ASPN*, *POSTN*, *ADAM12*, and *TGFBI* (Fig. 4, E to H). The six genes are significantly up-regulated upon TGF-β1 stimulation and counter-modulated in CRISPR-Cas9 *RPTOR* gene-edited cells (table S2), suggesting that these genes form part of the mTORC1-dependent signature of *CTHRC1*^+^ fibroblasts.

To further determine whether the *CTHRC1*^+^ pathologic fibroblast population exhibits a TGF-β–dependent transcriptional signature, we performed Scissor analysis using an additional RNA-Seq dataset reported in the literature (*31*), consisting of four pHLF donor lines treated with and without TGF-β1 (*31*). This additional analysis confirmed our finding that the Scissor^−^ cells, mapping to the in vitro fibroblast population at baseline, closely matches that of freshly isolated fibroblasts from human lungs characterized by low expression of *CTHRC1* and *COL1A1.* By contrast, the transcriptomic signature of the corresponding pHLFs stimulated with TGF-β1 (Scissor^+^) overlaps with cells unique to IPF characterized by high expression of *CTHRC1* and *COL1A1* (fig. S6). Together, the Scissor analyses show that the TGF-β1 transcriptomic signature of cultured pHLFs using two independent scRNA-Seq datasets maps to a population of fibroblasts unique to IPF (high expression of *CTHRC1* and *COL1A1*) and further was observed across several pHLF donor lines cultured and exposed to TGF-β1 in two independent laboratories. To clarify if there is a functional role for CTHRC1 in regulating TGF-β1–induced collagen hypersynthetic phenotype, we used an siRNA approach to silence *CTHRC1* and found that siRNA-mediated knockdown of *CTHRC1* had no effect on baseline or TGF-β1–up-regulated procollagen production (fig. S7), indicating that *CTHRC1* expression is a marker of this population but does not regulate the acquisition of the ECM hypersynthetic phenotype in response to TGF-β1 stimulation.

### Selective mTORC1 inhibition prevents the acquisition of a collagen-high *CTHRC1*^+^ fibroblast phenotype following TGF-β1 stimulation

We next investigated if there was a direct functional link between mTORC1 signaling and the acquisition of the collagen-high *CTHRC1*^+^ phenotype in response to TGF-β1 stimulation by evaluating the effect of selective mTORC1 inhibition using the clinical stage bisteric mTORC1 inhibitor, RMC-5552, which was developed in the oncology setting and which, to the best of our knowledge, has not been examined in the context of fibrosis.

**Fig. 5.**
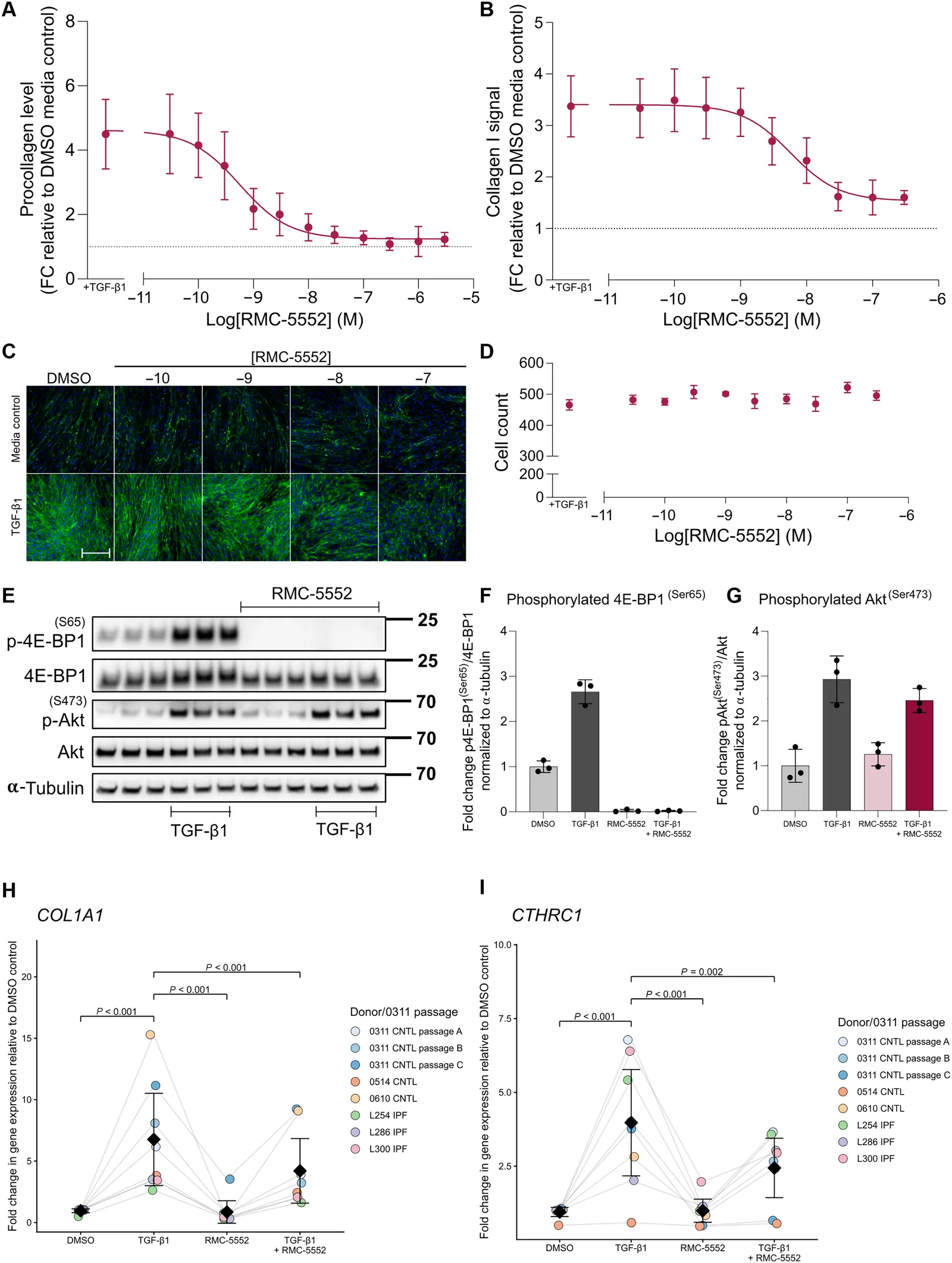
mTORC1-selective inhibitor RMC-5552 inhibits the TGF-β1–induced fibrotic response. pHLFs were exposed to RMC-5552 (0.01 μM) or vehicle control media with or without TGF-β1 (1 ng/ml) for 24 hours. (**A**) Procollagen data based on hydroxyproline levels in secreted proteins are expressed as fold change relative to vehicle control media. Data points represent the means ± SD for three independent experiments (passages) conducted on one control donor line. IC_50_ = 5.658 × 10^−10^ M [95% confidence interval (CI) = 2.383 × 10^−10^ to 1.388 × 10^−9^ M, estimated by four-parameter nonlinear regression]. FC, fold change. (**B**) Collagen I deposition assessed by high-content imaging for fibroblasts cultured in macromolecular crowding conditions. Data points represent the means ± SD for three control donor lines and are expressed as fold change in collagen I signal relative to control media. IC_50_ = 5.708 × 10^−9^ M (95% CI = 2.259 × 10^−9^ to 1.970 × 10^−8^ M; estimated by four-parameter nonlinear regression). (**C**) Representative images for each treatment group for (B). Scale bar, 360 μm. (**D**) Cell counts at the end of the experiment. (**E**) Western blot showing specified proteins for one experiment. Images for additional independent experiments (passages) performed with the same donor line are shown in fig. S9. Marker positions correspond to molecular weight (kDa). Nitrocellulose membranes are shown in fig. S10. (**F** and **G**) Densitometry for phosphorylated proteins with data expressed as means ± SD from three independent experiments (passages) of the same control donor line. (**H** and **I**) Fold change in expression of the genes (H) CTHRC1 and (I) COL1A1 in multiple control and IPF fibroblast lines. Expression is relative to GAPDH. Data points represent the means for three technical replicates (wells) for each donor line or passage. Statistical analysis was performed using a linear mixed-effects model. *P* values are based on ΔCt values (see fig. S11).

The evaluation of the effect of RMC-5552 on the TGF-β1–induced collagen response revealed that RMC-5552 inhibited TGF-β1–induced procollagen synthesis (based on hydroxyproline levels) at subnanomolar concentrations [median inhibitory concentration (IC_50_) = 5.658 × 10^−10^ M (95% confidence interval = 2.383 × 10^−10^ to 1.388 × 10^−09^ M) (Fig. 5A) in pHLFs (Fig. 5A). We also examined the effect of RMC-5552 on TGF-β1–induced collagen deposition in macromolecular crowding conditions in additional fibroblast lines derived from control lungs and from patients with IPF and from patients with lung adenocarcinoma (CAFs). All cell lines were responsive to TGF-β1, and this response was inhibited in a concentration-dependent manner in cells exposed to RMC-5552. IC_50_ values for the inhibitory response were similar across control, IPF, and CAF fibroblasts (Fig. 5, B to D, and fig. S8). Consistent with this compound acting via selective inhibition of mTORC1, Western blot analysis demonstrated that RMC-5552 inhibited TGF-β1–induced 4E-BP1 phosphorylation and had no impact on the phosphorylation of the mTORC2 substrate, Akt (Ser^473^) (Fig. 5, E to G, and fig. S9). We next examined the effect of this inhibitor on TGF-β1–increased *COL1A1* and *CTHRC1* mRNA levels in multiple fibroblast lines derived from control lungs and from patients with IPF and found that RMC-5552 significantly inhibited these transcriptional responses at the lowest concentration required to inhibit mTORC1 activity (Fig. 5, F and G). Together, these data support a key role for the TGF-β1/mTORC1 axis in defining the transcriptional identity of the collagen-high *CTHRC1*^+^ fibroblast population.

## DISCUSSION

**Fig. 6.**
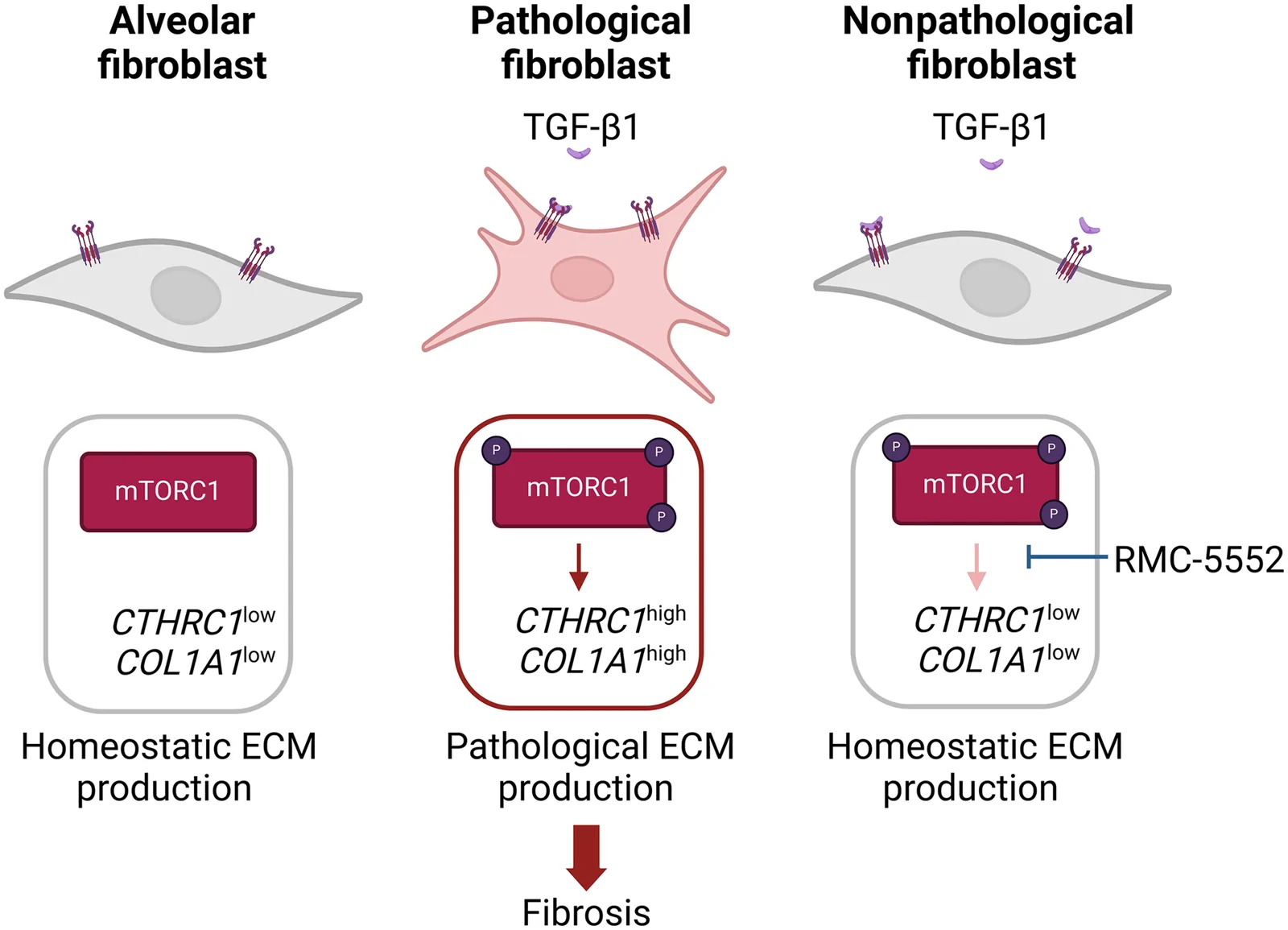
Proposed model of the functional link between mTORC1 and the regulation of key TGF-β1–up-regulated genes, which define the transcriptional identity of the *CTHRC1*^+^ fibroblast population in fibrosis. TGF-β1 activates quiescent fibroblasts and the mTORC1 axis. mTORC1 controls the up-regulation of key genes that define the transcriptional identity of the *CTHRC1*^+^ fibroblast population, which plays a key role in pathological ECM production and resulting fibrosis. The mTORC1-selective inhibitor, RMC-5552, blocks this response, highlighting mTORC1 as a potential antifibrotic target.

The cardinal profibrotic mediator TGF-β1 has been widely implicated in promoting fibrogenesis in multiple fibrotic conditions. Therapeutic strategies aimed at targeting the TGF-β1 axis in fibrosis, without compromising its critical roles in tissue and immune homeostasis, are therefore actively being pursued. We have previously reported that in addition to the canonical Smad signaling pathway, TGF-β1–induced collagen I production is under critical regulatory control by the mTORC1/4E-BP1 signaling hub and further that mTOR signaling acts, at least in part, by influencing *COL1A1* mRNA levels (*16*). TGF-β1 activates the Smad3 pathway that influences early levels of *COL1A1* mRNA and also activates the mTORC1/4E-BP1 axis, which consequently regulates maximal levels of *COL1A1 mRNA* (*16*). We previously also identified a key role for mTORC1 in the amplification of the ATF4-dependent de novo serine-glycine pathway to supply glycine during TGF-β1–induced collagen synthesis (*21*). We now report that the role of the mTORC1 axis extends to the regulation of over a third of all TGF-β1–regulated matrisome/matrisome-associated genes and further that the global transcriptome of TGF-β1–stimulated fibroblasts matches that of the collagen-high *CTHRC1*^+^, fibroblast population recently identified in the fibrotic lung (*8*). In contrast, the TGF-β1 induced transcriptome of fibroblasts, in which mTORC1 signaling is disrupted, does not map to any known fibroblast population. Last, using the clinical stage bisteric mTORC1-selective inhibitor from the oncology setting, RMC-5552, we provide evidence for a direct functional link between mTORC1 and the regulation of TGF-β1–induced *COL1A1* and *CTHRC1* gene expression, two key characteristics that define the pathological *CTHRC1*^+^ fibroblast population in the setting of IPF (Fig. 6). We further demonstrate that RMC-5552 exerts potent antifibrotic effects in IPF fibroblasts and in CAFs derived from patients with lung adenocarcinoma.

The ECM is composed of a complex array of multidomain macromolecules, which are organized in a tissue-specific manner to form a structurally stable network that determines the mechanical properties of tissues. The ECM also acts as a reservoir of growth factors and bioactive molecules. Biochemical and biomechanical signals received from the ECM direct cellular function and differentiation and play a decisive role during development and the maintenance of tissue homeostasis. The healthy ECM is maintained by the activity of resident fibroblasts and comprises a loose meshwork of collagens, elastin, and fibronectin and other core matrisome proteins. During pathological fibrosis, fibroblasts transdifferentiate into activated fibroblasts, which synthesize high levels of ECM molecules and markedly increase ECM and tissue stiffness by enzymatic covalent crosslinking of collagen and elastin. The profound influence of TGF-β1 on matrisome and matrisome-associated gene expression reported here was not unexpected, but the observation that more than a third of the TGF-β1–induced ECM transcriptional response is under mTORC1 regulatory control is notable. In contrast, mTORC2 is largely redundant for the TGF-β1–induced global transcriptional response in human lung fibroblasts. We show that the mTORC1-regulated ECM transcriptional program includes numerous core matrisome genes encoding fibrillar and nonfibrillar collagens, elastin, proteoglycans, and other ECM-associated proteins and processing enzymes. Also included are a number of genes encoding glycoproteins that can modulate growth factor signaling, such as fibronectin and members of the fibrillin and latent TGF-β binding protein (LTBP) families that form a microfibrillar network, which not only enhances the structural integrity of the ECM but also influences the binding of growth factors (*32*–*34*). We also identify putative transcription factors predicted to regulate the TGF-β1/mTORC1-dependent transcriptome, including Smad3 and ATF4, already strongly associated with TGF-β1 signaling and ECM production (*14*, *21*–*23*). We encourage readers to visit our interactive table S1 as a resource to run future queries on the transcriptomic datasets.

In the specific setting of IPF, the histological hallmark of this condition, the fibrotic focus, comprises dense aggregations of activated fibroblasts embedded in a collagen-rich ECM. Recent scRNA-Seq studies in IPF have uncovered a large degree of transcriptional heterogeneity among fibroblasts (*8*, *24*), with current evidence indicating the presence of multiple fibroblast populations with unique transcriptomic signatures. These include alveolar fibroblasts, *ACTA2*-positive myofibroblasts, *HAS1*-high fibroblasts, *PLIN2*-positive fibroblasts, and *CTHRC1*-expressing fibroblasts (*7*, *8*). Although the relative contribution of these populations to the fibrotic response remains to be fully elucidated, recent evidence indicates that α-SMA is not specific to pathologic ECM-producing fibroblasts (*8*). In contrast, *CTHRC1* was identified as a more specific marker of the small subset of fibroblasts that produce the highest levels of ECM proteins in pulmonary fibrosis and other fibrotic diseases (*8*). Our analysis using existing IPF datasets is consistent with these observations and shows the highest *COL1A1*- and *CTHRC1*-expressing fibroblast populations are present in IPF patient lungs, but not in healthy control lungs. The *COL1A1^+^* and *CTHRC1*^+^ fibroblast population were previously reported to be localized within the collagen-rich fibrotic foci in IPF and *Cthrc1^+^* fibroblasts isolated from the lungs of bleomycin-injured mice further exhibit a greater migratory potential, suggesting a role for *CTHRC1*^+^ fibroblasts in the progression of pulmonary fibrosis (*8*). This fibroblast population has also been identified in other fibrotic conditions, including fibrosis affecting the heart, liver, and skin and constitutes a major cellular target for the development of innovative anti-fibrotic therapeutic approaches (*35*–*38*). While the cellular origin of these subpopulations of fibroblasts in human fibrotic lung disease remains at present elusive, recent lineage-tracing studies in the bleomycin model of lung fibrosis revealed that alveolar fibroblasts represent the main source of multiple fibroblast populations, with the collagen- high *CTHRC1*^+^ fibroblast likely arising from this cell type in response to TGF-β1 signaling within the fibrotic niche (*25*). In mouse lungs, Mayr and colleagues describe a transitional fibroblast cell characterized by high Sfrp1 expression, which appears early after injury in peribronchiolar, adventitial and alveolar locations, and differentiate into Cthrc1^+^ fibroblasts (*30*). In pHLFs, TGF-β1 suppresses SFRP1 and induces their switch to an invasive *CTHRC1*^+^ myofibroblast identity (*30*). Our analyses of single-cell populations most closely resembling untreated fibroblasts grown in vitro from lung explants, revealed that these cells present markers of both alveolar and adventitial identity and the highest expression of *SFRP1* compared to all other lung fibroblasts.

Our understanding of the function and role of CTHRC1 during wound repair and fibrosis is at present incomplete. However, CTHRC1 was originally identified to play an essential role in reestablishing and maintaining tissue homeostasis after wound closure by modulating both the TGF-β and canonical Wnt signaling pathways [reviewed in (*15*)]. This glycoprotein has also been reported to play a protective role based on bleomycin studies in *Cthrc1*^−^ knockout mice (*39*), although the current body of evidence points overwhelmingly to a pathological role for the *CTHRC1*^+^ fibroblast population during fibrogenesis in multiple organs and tissues (*38*).

The integration of the TGF-β–regulated transcriptome in human lung fibroblasts using the bioinformatic tool Scissor (*26*), with two existing IPF scRNA-Seq datasets revealed a key role for mTORC1 signaling in defining the transcriptomic profile of the collagen-high *CTHRC1*^+^ fibroblast population. We further describe key genes and pathways defining the *CTHRC1*^+^ population with the top pathways affected in *CTHRC1*^+^ fibroblasts found to be related to ECM organization and function. *COL1A1* is expectedly one of the most positively regulated genes within this population but to the best of our knowledge *ASPN*, *ADAM12* and *TGFBI* have not been previously reported to be highly expressed by the pathologic *CTHRC1*^+^ fibroblast population. A final key gene, *POSTN,* was recently shown to be highly expressed in *Cthrc1*^+^ mouse fibroblasts (*25*). *TGFBI* is a known TGF-β–regulated gene and encodes an RGD-containing protein, TGF-β–induced protein, that plays a role in cell-collagen interactions (*40*) and has been implicated in the pathogenesis of bleomycin-induced fibrosis (*40*). *POSTN* is a paralog of *TGFBI* and encodes periostin, which plays a multifaceted role in the pathology of fibrotic diseases across various organs, including fibrosis of the lung, heart, liver, and kidney by modulating cell adhesion, migration, ECM remodeling, inflammation, and fibroblast activation. Moreover, previous work from our laboratory and others has shown that *POSTN* is highly expressed in IPF fibrotic foci and in the bleomycin model of fibrotic lung injury (*41*, *42*). *ASPN* encodes the proteoglycan asporin that has also been reported to be highly up-regulated in the lungs of IPF patients (*43*) and has been found to be localized to *ACTA2*-positive myofibroblasts (*44*). Asporin influences recycling of the TGF-β receptor and increases its stability at the cell surface. The knockdown of this gene suppresses TGF-β1 signaling, which is believed to be due to the loss of this recycling mechanism. It is therefore tempting to speculate that an increase in the expression of *ASPN* in *CTHRC1*^+^ fibroblasts might prolong their responsiveness to TGF-β1 and promote a positive feed-forward loop to drive fibrogenesis to TGF-β1 within the fibrotic niche. *ADAM12* encodes disintegrin and metalloproteinase domain-containing protein 12 and has been implicated in a variety of cellular processes involving cell-cell and cell matrix interactions. Previous lineage tracing studies in mice have shown that *ADAM12^+^* cells represent transient precursors to profibrotic cells that produce excessive levels of collagen following tissue injury (*45*), but the relevance of this observation to human disease remains to be established.

The data presented here uncover a critical role for the mTORC1 axis in influencing the transcriptional identity of the *CTHRC1*^+^ high collagen–expressing pathological fibroblast population induced in response to TGF-β1 signaling within the fibrotic niche. This observation is consistent and extends our previous finding obtained by transcriptome analysis of IPF lung tissue, which revealed that both TGF-β1 and the upstream regulator of mTORC1 activation (TSC2/RHEB) formed major signaling clusters associated with collagen gene expression in IPF fibrotic foci captured by laser capture microdissection (*41*). The importance of mTORC1 signaling in IPF is further supported by genetic association studies. A recent meta-analysis of genome-wide association analysis (GWAS) of five IPF cohorts identified *DEPTOR*, which encodes a major endogenous regulator of mTOR signaling, as a candidate IPF susceptibility gene. The IPF risk allele is predicted to be associated with reduced expression of DEPTOR and potentially uncontrolled mTOR signaling (*46*).

Our observations have important therapeutic implications in terms of targeting the CTHRC1^+^ fibroblast population and suppressing excessive ECM deposition in IPF and potentially multiple fibrotic conditions. Therapeutic strategies targeting both the TGF-β1 and mTOR signaling axes have been intensely pursued for over two decades, but their clinical success has been hampered by the key roles these signaling axes play in immune homeostasis and tumor suppression in the case of TGF-β1, whereas ATP competitive dual mTOR inhibition leads to undesirable effects on glucose metabolism and relief of AKT-dependent feedback inhibition of RTK expression as a result of concomitant mTORC2 inhibition (*19*). The recent development of selective bisteric inhibitors of mTORC1 now offers the potential to overcome some of the major hurdles encountered with earlier mTOR targeting approaches. These mTORC1 selective inhibitors potently inhibit p70S6K and 4E-BP1 phosphorylation while limiting unwanted effects as a result of concomitant mTORC2 inhibition (*19*). At the time of writing, the lead compound, RMC-5552, used in this study was being evaluated in clinical trials in the oncology setting (*20*). The scientific rationale for evaluating the antifibrotic potential of selective mTORC1 inhibition in the setting of fibrosis and cancer is gaining strength.

Several limitations should be acknowledged. First, scissor analysis using fibroblasts derived from multiple IPF donors and integrated with IPF scRNA-seq data would have been informative, but the marked heterogeneity of patient-derived fibroblast lines means that such an analysis would require a larger panel of IPF donor lines than was available for this study. This does not undermine our principal conclusion that the mTORC1 axis shapes TGF-β1–induced fibroblast transcriptional responses but instead identifies an important area for future investigation. Second, we addressed the reproducibility of the impact of mTORC1 inhibition on the expression of the key marker genes, *COL1A1* and *CTHRC1*, following TGF-β1 activation across multiple control and IPF fibroblast lines (*n* = 6) using a linear mixed-effects model (LMM) framework; nevertheless, evaluation in a larger number of lines would further support the generalizability of these findings. Last, in vitro work was restricted to fibroblasts cultured in isolation. Confirmation in more complex multicellular models would provide additional translational support for selective mTORC1 inhibition as a therapeutic approach.

## MATERIALS AND METHODS

### Primary cell culture

pHLFs from healthy donors and IPF patients and CAFs were isolated from lung tissue and expanded as previously described (*16*). All cells were cultured at 37°C in 5% CO_2_ in either Dulbecco’s modified Eagle’s medium (Sigma-Aldrich) (Figs. 1 to 4) or Plasmax (Cancer Tools) (Fig. 5). For cell growth, the medium was supplemented with 10% fetal bovine serum (FBS) and 1% penicillin streptomycin (both Sigma-Aldrich) unless stated otherwise. All experiments were carried out on cells between passages 2 and 8.

For TGF-β1 stimulation, cells were exposed to TGF-β1 (1 ng/ml; Bio-techne) for 24 to 48 hours depending on the downstream assay. For RMC-5552 exposure, RMC-5552 (HY-132168, MedChem Express) was resuspended in 1 mM dimethyl sulfoxide (DMSO) and diluted to the required concentrations using physiologically relevant media supplemented with 0.4% FBS (Plasmax, Cancer Tools). TGF-β1–stimulated or media treated cells were exposed to media supplemented with either RMC-5552 or DMSO for 48 hours.

### CRISPR-Cas9 gene editing

CRISPR-Cas9 gene editing was carried out in pHLFs according to methods described previously (*16*). Guide RNA sequences were designed using the Deskgen design platform and are outlined in Table 1. Matched wild-type controls were generated using Alt-R Negative Control crRNA 1 (2 nmol; IDT).

**Table 1.** Primers for CRISPR-Cas9 gene editing and qPCR. Rev., reverse. Fwd, forward. gRNA, guide RNA.

Gene	Primer
CRISPR-Cas9 gene editing	
*RICTOR*	
gRNA	AATATCGGCTCATCAAATTGGGG
MiSeq For	ACACTCTTTCCCTACACGACGctcttccgatctATGACCTACCCTCTGATGGAAAG
MiSeq Rev	TGACTGGAGTTCAGACGTGTGctcttccgatctTTTTTCTCTCTCAGAGATGAGGT
*RPTOR*	
gRNA	CCGCGTCTACGACACAGAAGGATGG
MiSeq For	ACACTCTTTCCCTACACGACGctcttccgatctACCCAACCAAATGGCAGTGACAC
MiSeq Rev	TGACTGGAGTTCAGACGTGTctcttccgatctTGCCTGTGTTTGGCTCTAGGACA
qPCR	
*COL1A1* For	ATGTAGGCCACGCTGTTCTT
*COL1A1* Rev	GAGAGCATGACCGATGGATT
*CTHRC1* For	GCCATGCGACCCCAGG
*CTHRC1* Rev	ACTCCTGCTGGCCCTTGTAA
*GAPDH* For	TCCTGTTCGACAGTCAGCCG
*GAPDH* Rev	CCCCATGGTGTCTGAGCGAT

### RNA sequencing

The RNA-Seq datasets described in Fig. 1 (GSE102674) were generated as previously described (*21*). To generate the RNA-Seq datasets described in Fig. 2, RNA was extracted using the RNeasy Mini Kit (QIAGEN), and 500 ng of RNA was used as input for polyadenylate-tailed RNA enrichment and library preparation using the KAPA mRNA HyperPrep Kit (Kapa Biosystems). The assessment of library quality and quantification was carried out using Bioanalyzer High Sensitivity DNA analysis and the Qubit High Sensitivity fluorometry assay according to the manufacturer’s instructions. Paired-end sequencing was performed on the NextSeq sequencing platform (Illumina). The generation of the RNA-Seq dataset described in fig. S6 is outlined in (*31*).

### RNA-Seq analysis

The sequencing data were uploaded to the Galaxy web platform (*47*), where sequenced reads were quality tested using FastQC and MultiQC (*48*, *49*) aligned to the hg38 human genome using HISAT2 with default parameters (*50*). The quantification of counts over reference genes was then performed using FeatureCounts (*51*). The differential gene expression analysis was carried out using DESeq2 (*52*) within R/RStudio (*53*, *54*). We applied a standard interaction design formulae of design ∼ genotype + condition + genotype:condition to take into account baseline transcriptomic differences introduced by drug treatment or gene editing and to identify condition-dependent effects beyond those baseline changes. DEGs were defined as having an adjusted *P* value < 0.05 and fold change > 1.5 or < −1.5 when comparing two experimental conditions. The ClusterProfiler package was used within R/RStudio to conduct overrepresentation pathways analysis using the Reactome database (*55*, *56*), and significant pathways were defined as having an adjusted *P* value <0.05. The QIAGEN IPA URA tool was used with default settings and Molecule Type was limited to transcriptional regulators only. Data visualization was carried out using ClusterProfiler, as well as EnhancedVolcano (*57*) and pheatmap (*58*).

### Scissor analysis

The processed scRNA-Seq gene expression matrices were extracted from the Gene Expression Omnibus (GSE136831 and GSE135893). For GSE136831, normalized counts for cells labeled as fibroblasts or myofibroblasts by Adams *et al.* (*24*) were extracted. For GSE135893, normalized counts for cells labeled as fibroblasts, *HAS1* high fibroblasts, myofibroblasts, and *PLIN2^+^* fibroblasts were extracted. The Seurat preprocessing function within the single-cell identification of subpopulations with bulk sample phenotype correlation (Scissor) R package was used to preprocess the data and create a cell-cell similarity network (*26*). The Scissor algorithm was then applied to identify individual cells within the scRNA-Seq dataset with expression profiles that are correlated with the expression profile of control media or TGF-β–stimulated samples from the bulk RNA-Seq datasets. The alpha parameter of 0.5 was used for which the reliability significance test reported *P* < 0.001. The FindAllMarkers function in Seurat was used to identify DEGs for the Scissor^+^ and Scissor^−^ cells versus all other cells (*P* value < 0.05 and fold change > 1.5 or < −1.5) (*59*). Pathways analysis was carried out using ClusterProfiler as described above (*55*, *56*).

### siRNA transfection

Primary HLFs were transfected with 20 nM ON-TARGETplus siRNA pool targeting *CTHRC1* or a negative control small interfering RNA (siRNA) (Thermo Fisher Scientific) using RNAiMax Lipofectamine (Invitrogen) according to the manufacturer’s instructions.

### Real-time quantitative PCR

Total RNA extraction was carried out using the RNeasy Mini Plus kit (QIAGEN) according to the manufacturer’s instructions. cDNA synthesis was carried out with 500 ng of total RNA input using the qScript cDNA Supermix kit (Quanta Biosciences) according to the manufacturer’s instructions. Quantitative polymerase chain reaction (qPCR) was performed using the Power SYBR Green PCR Master Mix (Thermo Fisher Scientific) with 5 μl of SYBR green PCR mix per 2 μl of cDNA sample. Forward and reverse primers were added at a final concentration of 800 nM, and sequences of primers used are outlined in Table 1. qPCR was performed on the QuantStudio 5 platform (Thermo Fisher Scientific) with the following cycling conditions for 40 cycles: 95°C for 10 min, 95°C for 15 s, and 60°C for 1 min. The Ct values were normalized to the housekeeping gene *GAPDH* (glyceraldehyde 3-phosphate dehydrogenase) by subtracting the average of the Ct values of the housekeeping genes. The relative expression of genes of interest was then calculated using the 2^-deltaCt^ approach.

### Immunoblotting

Total protein extraction was carried out using ice-cold PhosphoSafe Extraction Buffer (Merck Millipore) supplemented with a protease and phosphatase inhibitor cocktail (Roche). Protein concentration was quantified using a Pierce BCA Protein Assay kit (Thermo Fisher Scientific). Lysates were subjected to SDS–polyacrylamide gel electrophoresis reducing conditions using LDS sample buffer and reducing agent (Thermo Fisher Scientific). Lysates were run on Invitrogen Bolt 4-12% bis-tris gels (Thermo Fisher Scientific) at 160 V for 45 min and then transferred on to nitrocellulose membranes using an iBlot 2 Gel Transfer Device (Thermo Fisher Scientific) at 20 V for 7 min. Following transfer of proteins to nitrocellulose membranes, membranes were cut in two. The bottom section was probed for phosphorylated 4E-BP1, stripped with RestoreTM Western blot Stripping Buffer (Thermo Fisher Scientific), and probed for total 4E-BP1 or total Akt. This process was repeated for p-AKT and AKT on the top section, which was stripped again and probed for α-tubulin. Blocking and antibody washing were performed in 5% bovine serum albumin (Thermo Fisher Scientific) in tris-buffered saline with 0.1% Tween 20. Protein levels were assessed using the antibodies and dilutions highlighted in Table 2. Images were captured on an iBright 1500 (Thermo Fisher Scientific) and quantified with Thermo Fisher Scientific iBright Analysis Software.

**Table 2.** Antibodies and dilutions used in immunoblotting and macromolecular crowding assay.

Protein	Antibody	Manufacturer	Dilution
Phosphorylated 4E-BP1 (Ser^65^)	13443	Cell Signaling Technology	1:2000
4E-BP1 (total)	9644	Cell Signaling Technology	1:2000
Phosphorylated Akt (S473)	4060	Cell Signaling Technology	1:2000
Akt (total)	4691	Cell Signaling Technology	1:2000
Raptor	2280	Cell Signaling Technology	1:1000
Rictor	2114	Cell Signaling Technology	1:1000
α-Tubulin	9099	Cell Signaling Technology	1:2500
Collagen I	C2456	Cell Signaling Technology	1:1000
Alpha smooth muscle actin	M085101	Abcam	1:500
Alexa-Fluor 488	A11001	Thermo Fisher Scientific	1:1000
Alexa-Fluor 555	A21428	Thermo Fisher Scientific	1:1000

### Quantification of hydroxyproline from cellular supernatants

Procollagen production by pHLFs was assessed by quantification of hydroxyproline levels in the ethanol-insoluble fraction of secreted proteins by reverse-phase high-performance liquid chromatography as previously described (*16*).

### Macromolecular crowding assay

Collagen I deposition was measured by high-content imaging of cells grown in 96-well plates under macromolecular crowding conditions as described previously (*16*).

### Study approval

Human samples for the generation of control and IPF-derived pHLFs were acquired following research ethics committee approval (10/H0720/12, 10/H0504/9, and 12/EM/0058) and informed consent was obtained. pHLF cell lines were derived through explant culture, and their purity was confirmed by immunohistochemical characterization. Cancer-associated fibroblast (CAF) cell cultures were established within the Tracking Cancer Evolution through Therapy (TRACERx) clinical study (13/LO/1546—NRES London—Camden and Islington).

### Statistical analysis

All graphs were created in GraphPad Prism (version 10) or within R (v4.5.3) within the VScode (v1.111.0) or RStudio environments. Residuals are visualized by plotting the mean and the associated SD. Where specified in relevant figure legends, data were tested for normality using a Shapiro-Wilk test, then analyzed by one-way analysis of variance (ANOVA) with Dunnett’s multiple comparisons testing or two-way ANOVA with Holm-Šídák multiple comparisons test. Data were considered statistically significant at *P* < 0.05.

### Fitting of LMM to hierarchical data

For experiments involving multiple donors and multiple cell passages (Fig. 5, H and I, and see also fig. S11), statistical inference was performed using a LMM to account for the hierarchical structure of the data. A regression model of the form expression ∼ treatment + (1|donor) + (1|passage) was fitted to the raw delta cycle threshold (ΔCt) values, with donor and passage included as random intercepts to account for variability across donors and passages. Model assumptions were assessed by examining mean-normalized residuals and inspecting the variance at each level of the data hierarchy to evaluate approximate normality. The TGF-β1 treatment condition was specified as the reference baseline. Reported *P* values correspond to the probability of observing treatment effect estimates at least as extreme as those obtained for DMSO, RMC-5552, and TGF-β1 + RMC-5552 relative to the TGF-β1 baseline. All analyses and plots were generated in R (v4.5.3). LMMs were fitted using the lmer() function from the lme4 package (CRAN version 1.1-38).

## Supplementary Materials

### The PDF file includes:

Tables S1 to S3 Legends for tables S4 to S10 Figs. S1 to S11 References
