# Supplementary material for: Critical role for the TGF-β1/mTORC1 signaling axis in defining the transcriptional identity of CTHRC1+ pathologic fibroblasts
Source: Sci Adv. 2026 Sep 2;12(36):eadx9868. doi: 10.1126/sciadv.adx9868 (PMC13537237; doi:10.1126/sciadv.adx9868)
Supplement: Supplementary file 1 [file sciadv.adx9868_sm.pdf]

Science Advances
AAAS

Supplementary Materials for

Critical role for the TGF-β1/mTORC1 signaling axis in defining the transcriptional identity of CTHRC1$^+$ pathologic fibroblasts

Jo-Anne AM Wilson *et al.*

Corresponding author: Rachel C. Chambers, r.chambers@ucl.ac.uk

Sci. Adv. **12**, eadx9868 (2026)
DOI: 10.1126/sciadv.adx9868

The PDF file includes:

Tables S1 to S3
Legends for tables S4 to S10
Figs. S1 to S11
References

Other Supplementary Material for this manuscript includes the following:

Tables S4 to S10

Supplementary Tables

The supplementary tables can be found in the uploaded files entitled SupplementaryTables.html.

Table S1. Distribution of Scissor+ and Scissor- Cells according to Disease Status and Celltype Annotation. Scissor analysis performed with fibroblasts extracted from single cell RNA-Seq dataset GSE136831. Cell type annotations and disease status were taken from Adams *et al.* 2020 and overlaid onto the Scissor+ and Scissor- cells.

Cell Type	Percentage
**Scissor + Disease State**	
IPF	100%
Control	0%
**Scissor + Fibroblast Type**	
Fibroblast	11%
Myofibroblast	89%
**Scissor - Disease State**	
IPF	54%
Control	46%
**Scissor - Fibroblast Type**	
Fibroblast	90%
Myofibroblast	10%

Table S2. RNA-Seq differential gene expression results for the six genes with the highest positive fold changes that define the transcriptional identity of the *CTHRC1*+ fibroblast population. The six genes are significantly upregulated in pHLFs upon TGF-β1 stimulation (log2FC > 0.58 and adjusted p-value < 0.05), and significantly counter-modulated in CRISPR-Cas9 *RPTOR* gene-edited cells (log2FC < -0.58 and adjusted p-value < 0.05).

**Gene**	**TGF-β1 stimulation in CRISPR-Cas9 control cells**		**Counter-modulation in CRISPR-Cas9 *RPTOR* gene-edited cells**	
	**Log2FC**	**Adjusted p-value**	**Log2FC**	**Adjusted p-value**
ASPN	2.48	4.58E-231	-1.84	6.49E-64
POSTN	1.22	1.24E-84	-0.69	5.20E-14
CTHRC1	2.44	3.19E-271	-1.09	7.10E-29
ADAM12	4.06	0	-1.85	8.15E-157
TGFBI	3.59	0	-0.68	3.80E-65
COL1A1	2.12	0	-1.29	1.78E-96

Table S3. QIAGEN IPA Upstream Regulator Analysis predicts upstream regulators of the mTORC1-dependent transcriptome. The top 30 most significant upstream transcriptional regulators by p-value are shown.

Upstream Regulator	Molecule Type	p-value
TP53	transcriptional regulator	4.91E-38
ATF4	transcriptional regulator	2.68E-30
GLI1	transcriptional regulator	7.66E-25
MYC	transcriptional regulator	1.5E-19
TP73	transcriptional regulator	1.64E-19
CREB1	transcriptional regulator	3.31E-19
MRTFB	transcriptional regulator	6.49E-19
FOS	transcriptional regulator	1.01E-18
JUN	transcriptional regulator	3.87E-18
SMAD3	transcriptional regulator	5.64E-18
TP63	transcriptional regulator	1.8E-16
FOXO3	transcriptional regulator	2.09E-16
SREBF1	transcriptional regulator	2.67E-16
SMARCA4	transcriptional regulator	5.82E-16
CTNNB1	transcriptional regulator	7.94E-16
FOXO4	transcriptional regulator	1.09E-15
NPM1	transcriptional regulator	1.53E-15
STAT3	transcriptional regulator	1.79E-15
SP1	transcriptional regulator	2.18E-15
NUPR1	transcriptional regulator	4.13E-15
PPARGC1A	transcriptional regulator	3.65E-14
WT1	transcriptional regulator	4.59E-14
YAP1	transcriptional regulator	5.34E-14
HIF1A	transcriptional regulator	8.33E-14
TWIST1	transcriptional regulator	1.52E-13
EPAS1	transcriptional regulator	1.79E-13
FOXO1	transcriptional regulator	1.08E-12
MRTFA	transcriptional regulator	1.54E-12
NKX2-3	transcriptional regulator	9.19E-12
HTT	transcriptional regulator	9.59E-12

Tables S4-S6 - Pharmacological datasets - RNA-Seq data from pHLFs treated with AZD8055 or rapamycin

Tables S7-S8 - CRISPR/Cas9 datasets - RNA-Seq data from CRISPR/Cas9 gene edited pHLFs

Tables S9-S10 - Scissor analysis - mapping RNA-Seq data onto a human IPF scRNA-Seq dataset (GSE136831)

Fig. S1.

A

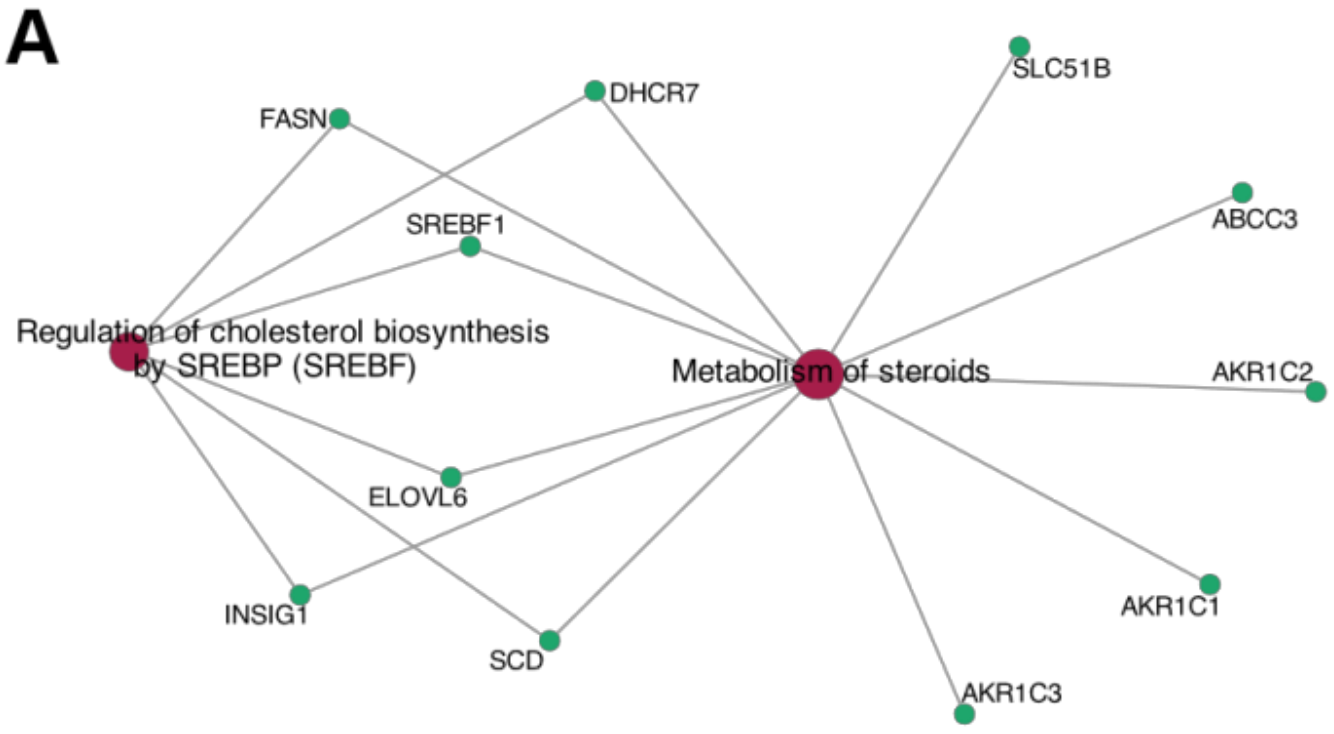

B

TYPE	GENES
ECM regulator	*ADAMTS3, ADAMTS6, TGM2*
Glycoprotein	*IGFBP5, IGSF10, SMOC1*
Proteoglycan	*FMOD*
Secreted factor	*ANGPTL4, FGF1, FGF9, FSTL3, GDNF, HGF, IGF1, IL11, NGF, NRG1*

Rapamycin has limited effects on the TGF-β1-induced transcriptional response in primary human lung fibroblasts. pHLFs were incubated with rapamycin (100 nM) for 1 hour prior to stimulation with or without TGF-β1 (1 ng/ml) for 24 hours and RNA-Seq was carried out. **(A)** Gene-connection network plot showing the only two significant Reactome pathways and their member genes (adjusted p-value < 0.01) from over-representation analysis of the rapamycin/TGF-β1-dependent genes. **(B)** Table showing the limited number of matrisomal genes counter-modulated by rapamycin (adjusted p-value < 0.05 and absolute fold change > 1.5).

Fig. S2.

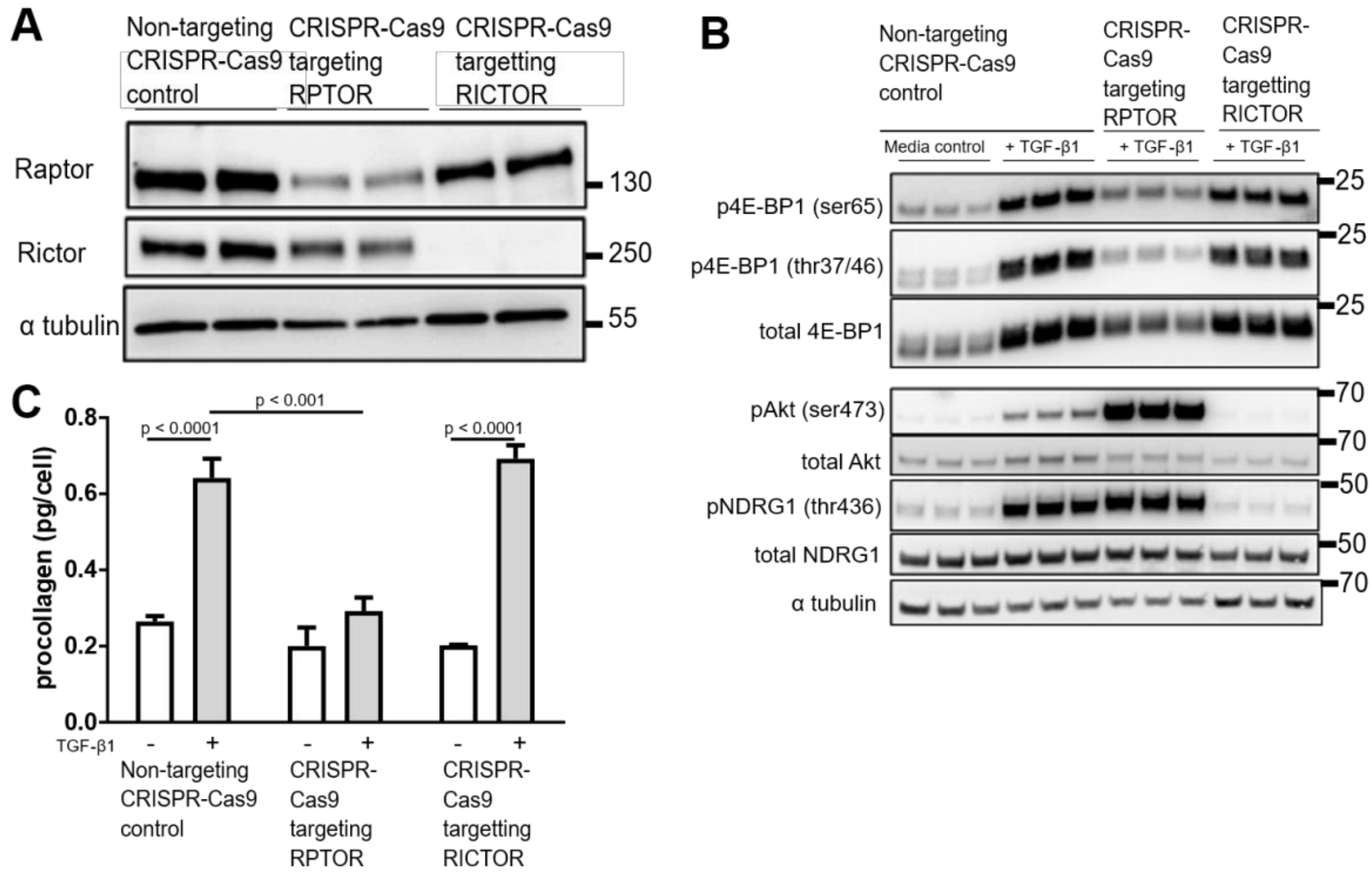

CRISPR-Cas9 gene editing targeting *RPTOR* but not *RICTOR* impacts TGF-β1-induced phosphorylation of the mTORC1/4E-BP1 pathway and hydroxyproline levels. pHLFs were modified by CRISPR-Cas9 gene editing using gRNAs targeting *RPTOR* or *RICTOR* and the cells were stimulated with or without TGF-β1 (1 ng/ml) for 24 hours. **(A, B)** Phosphorylation of specified proteins is shown by Western blot. **(C)** Procollagen levels are based on hydroxyproline levels in the ethanol-insoluble fraction of secreted proteins quantified by reverse-phase high-performance liquid chromatography. Each data point shown is mean ± SD (n = 3). Ordinary Two-way ANOVA statistical tests with Holm-Sidak multiple comparison testing was performed on C with statistical significance as $p<0.05$.

Fig. S3.

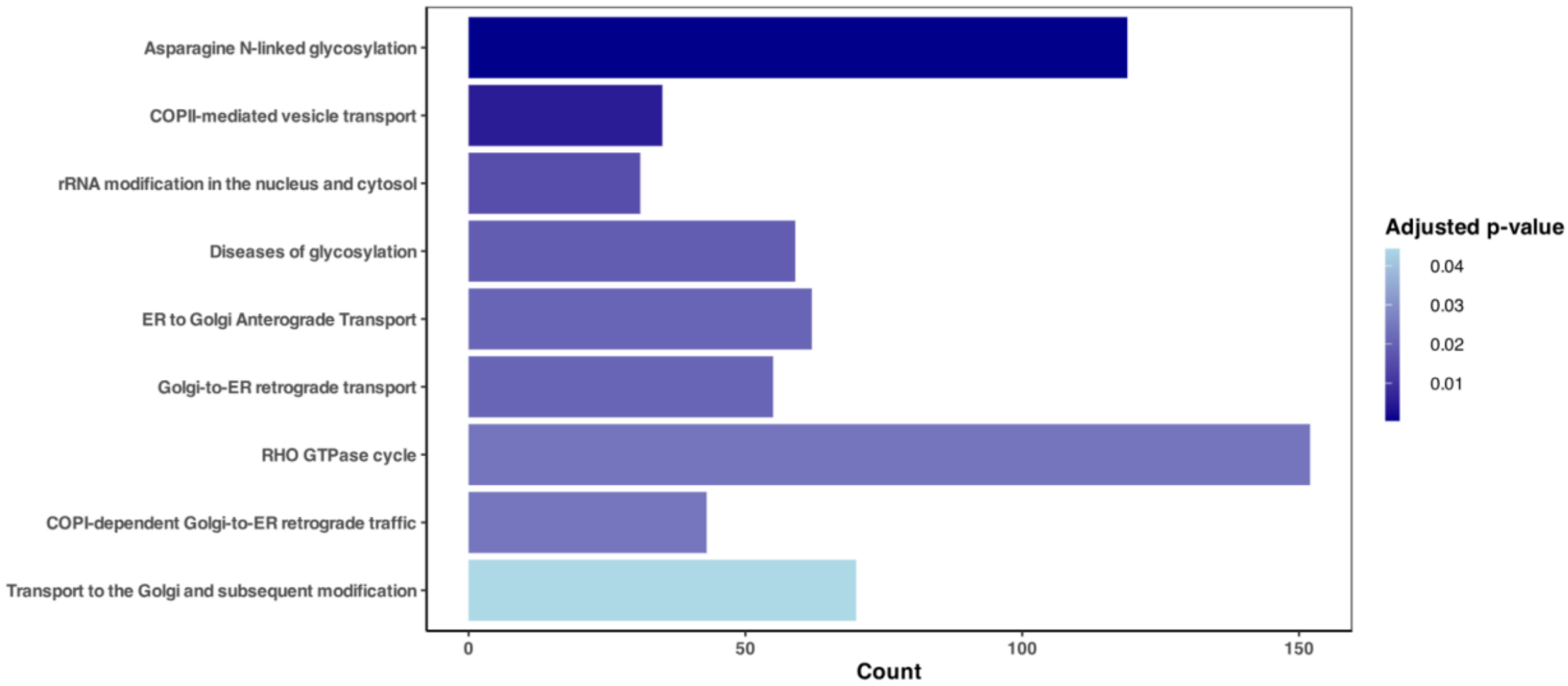

TGF-β1-dependent, mTORC1-independent genes impact the ER/Golgi secretory pathway, protein N-Glycosylation and RHO GTPase signaling. Bar-plot showing Reactome pathways (adjusted p-value < 0.05) from over-representation analysis of the 5684 TGF-β1-dependent, mTORC1-independent genes. The x-axis represents the number of genes enriched in that pathway and the color scale represents the adjusted p-value.

Fig. S4.

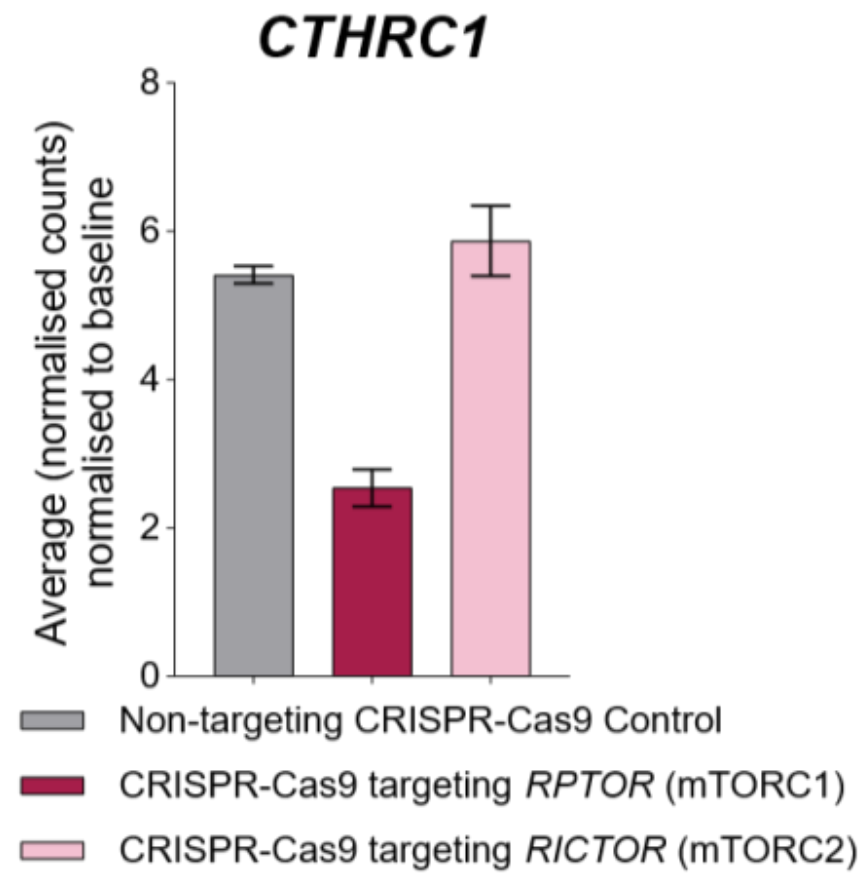

CRISPR-Cas9 gene editing targeting *RPTOR* but not *RICTOR* impacts TGF-β1-induced upregulation of *CTHRC1* expression. pHLFs were modified by CRISPR-Cas9 gene editing using gRNAs targeting *RPTOR* or *RICTOR* and the cells were stimulated with or without TGF-β1 (1 ng/ml) for 24 hours and RNA-Seq was carried out. Normalized counts for TGF-β1-stimulated samples were averaged across replicates and normalized to baseline. The fold change is plotted for CRISPR-Cas9 control cells, CRISPR-Cas9 *RPTOR* gene-edited cells and CRISPR-Cas9 *RICTOR* gene-edited cells.

Fig. S5.

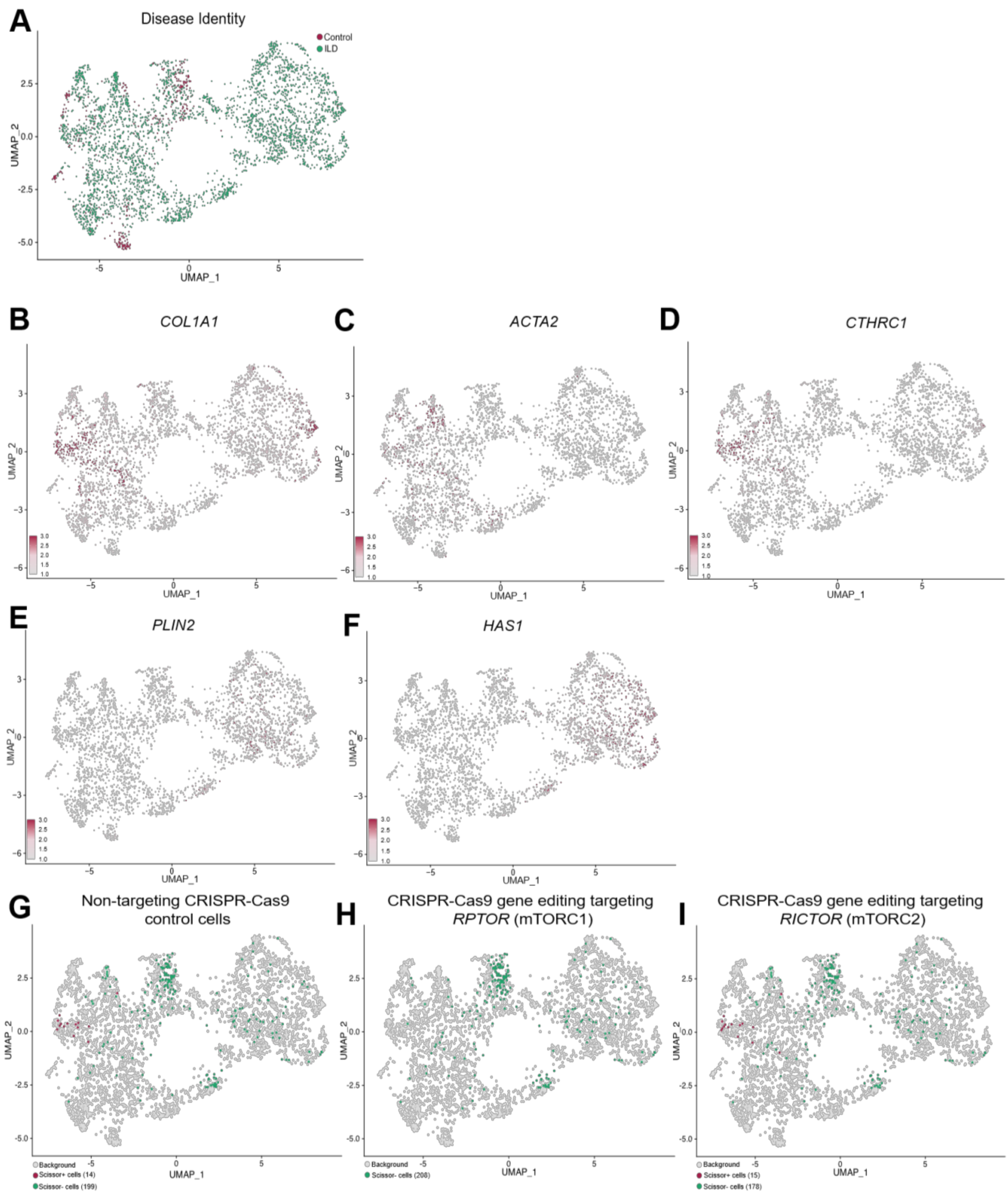

Integration of IPF scRNA-Seq and *in vitro* pHLF RNA-Seq data reveals a role for the TGF-β1/mTORC1 axis in promoting the appearance of a *CTHRC1*⁺ fibroblast population present in the IPF lung. Scissor was used to map bulk RNA-Seq data (from Figure 2) onto a human IPF scRNA-Seq dataset (GSE135893) (7). **(A)** UMAP visualization of the fibroblasts from the scRNA-

Seq dataset with the disease status of each cell shown. **(B-F)** UMAP visualization of the expression levels of key activated fibroblast markers from the literature including *COL1A1* **(B)**, *ACTA2* **(C)**, *CTHRC1* **(D)**, *PLIN2* **(E)** and *HAS1* **(F)**. UMAP visualization of the Scissor-selected cells for non-targeting CRISPR-Cas9 control cells **(G)**, CRISPR-Cas9 *RPTOR* gene-edited cells **(H)**, and CRISPR-Cas9 *RICTOR* gene-edited cells **(I)**, where the maroon and green dots are cells associated with TGF-β1-stimulated (Scissor+) and media control (Scissor-) cells, respectively, while background cells are grey.

Fig. S6.

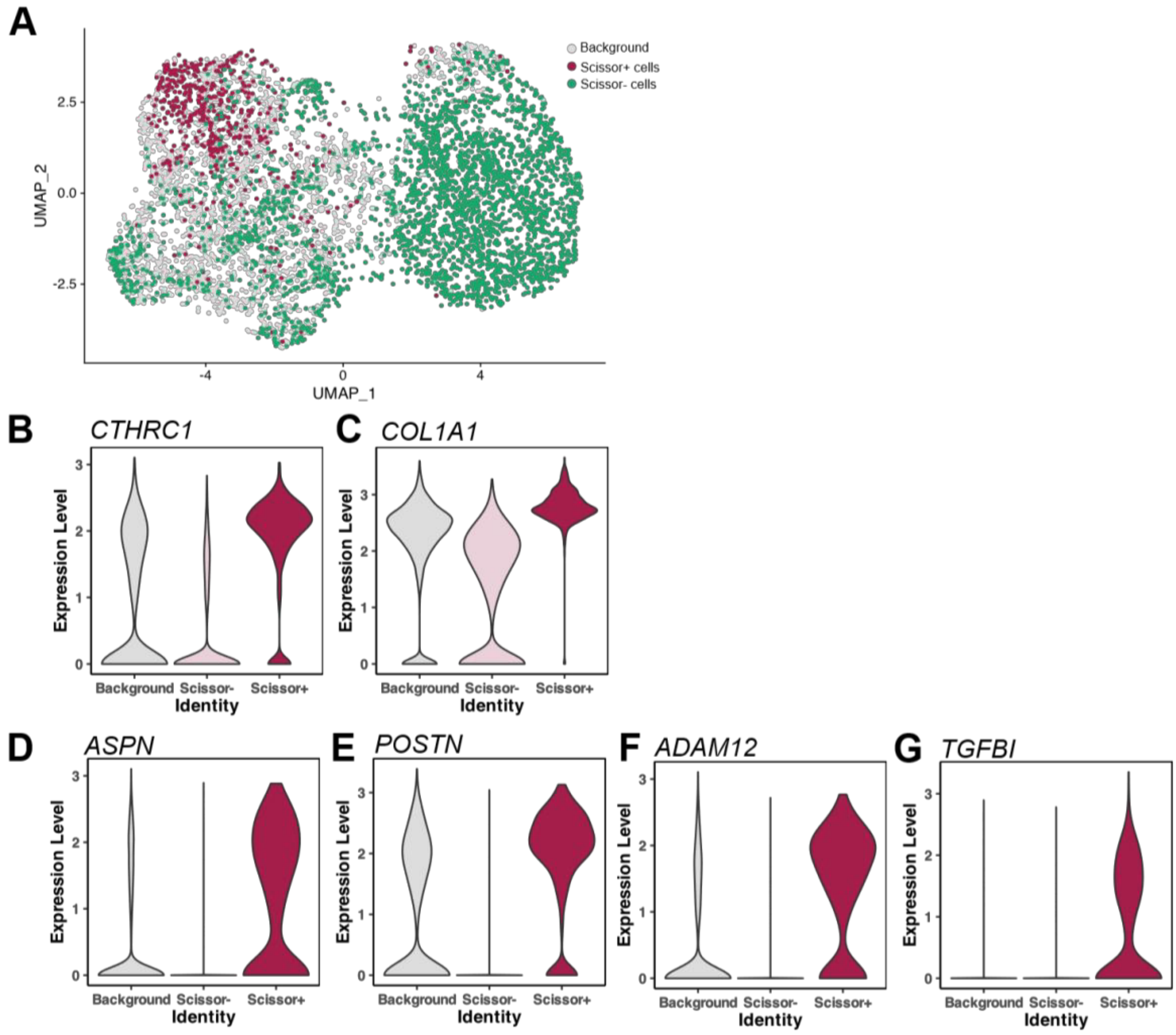

Integration of IPF scRNA-Seq and *in vitro* TGF-β-stimulated pHLF RNA-Seq data reveals a role for the TGF-β axis in promoting the appearance of a *CTHRC1*⁺ fibroblast population related to IPF. Scissor was used to map bulk previously published RNA-Seq data from four fibroblast donor lines stimulated with TGF-β (31) onto a human scRNA-Seq dataset (GSE136831). **(A)** UMAP visualization of the Scissor-selected cells where the maroon and green dots are cells associated with TGF-β-stimulated (Scissor+) and media control (Scissor-) cells, respectively, while background cells are grey. **(B-G)** Violin plots show the expression of the six genes, (**C**) *CTHRC1* (**D**) *COL1A1* (**E**) *ASPN* (**F**) *POSTN* (**G**) *ADAM12* and (**H**) *TGFBI,* in the Scissor+ population (maroon) with the highest fold changes in comparison to the Scissor- (light pink) and all other fibroblasts in the dataset (grey).

Fig. S7.

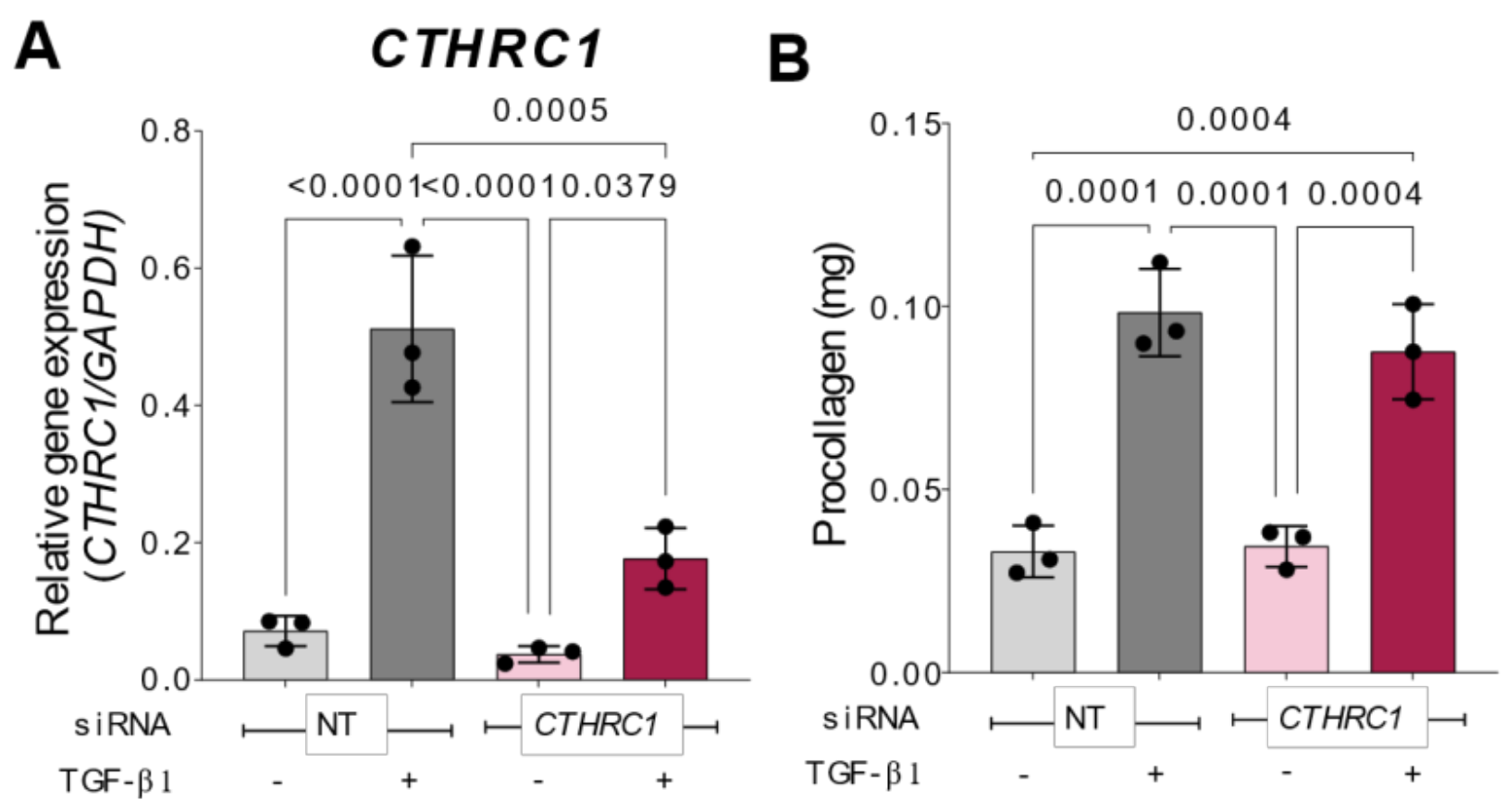

Knockdown of *CTHRC1* does not impact the TGF-β1-induced collagen response. pHLFs were exposed to siRNA mediated knockdown of *CTHRC1* or a non-targeting control and were stimulated with or without TGF-β1 (1 ng/ml) for 24 hours prior to experimental analysis. **(A)** Relative gene expression of the *CTHRC1* are shown by qPCR. Expression is relative to the housekeeping gene GAPDH. **(B)** Procollagen levels at 24 hours are based on hydroxyproline levels in the ethanol-insoluble fraction of secreted proteins quantified by reverse-phase high-performance liquid chromatography. Data in A and B represent mean ± SD from n = 3 independent experiments conducted on 3 control donor lines. Ordinary Two-way ANOVA statistical tests with Holm-Sidak multiple comparison testing was performed on A and B with statistical significance as $p<0.05$.

Fig. S8.

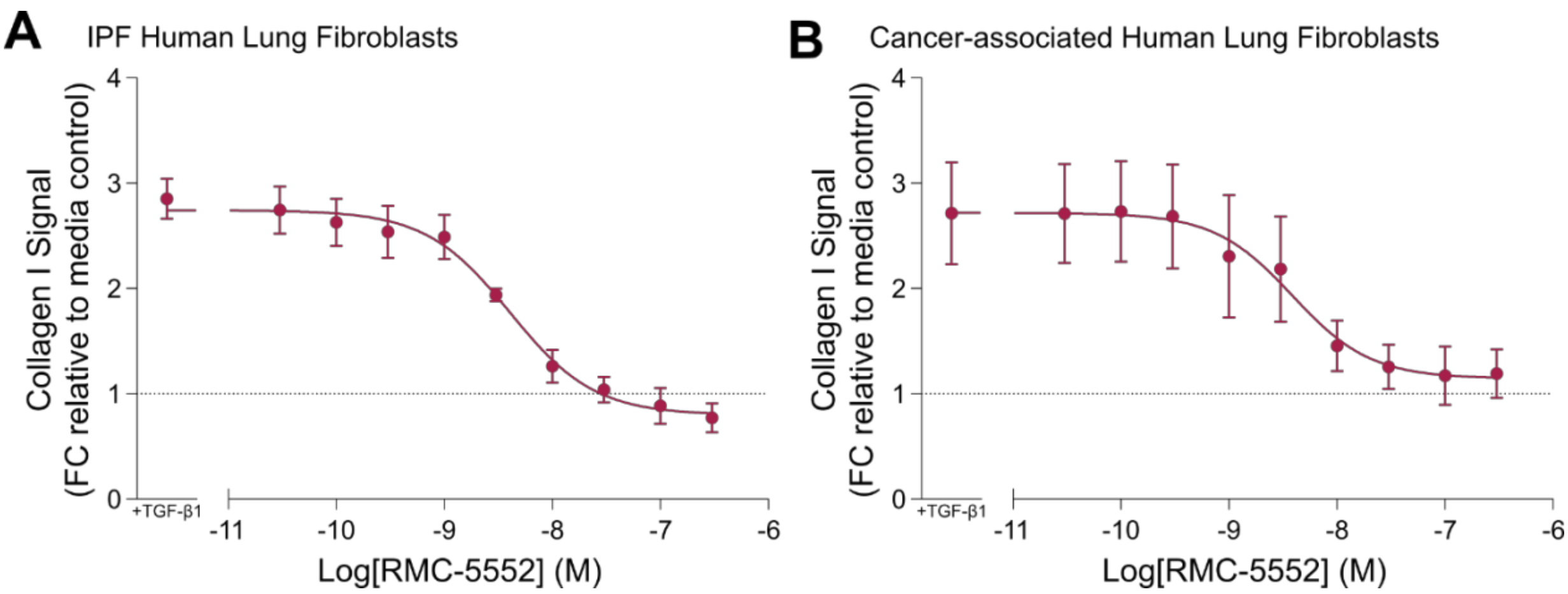

mTORC1-specific inhibitor RMC-5552 inhibits the TGF-β1-induced fibrotic response in multiple lung fibroblast lines. pHLFs were exposed to the mTORC1-selective inhibitor RMC-5552 or drug vehicle (DMSO) control and were stimulated with or without TGF-β1 (1 ng/ml) for 24 hours prior to experimental analysis. **(A, B)** Collagen I deposition assessed by high-content imaging of cells grown in macromolecular crowding conditions from (**A**) IPF patients (IC50 = 2.464 x10-9 M (95% CI = 1.255 x10-9 M to 4.384 x10-9 M)) and (**B**) cancer-associated fibroblasts (IC50 = 2.620 X10-9 M (95% CI = 1.563 X10-9 M to 4.298 x10-9 M). Data are expressed as the fold change in collagen I signal relative to the media control from 3 individual donor lines for each lung condition.

Fig. S9.

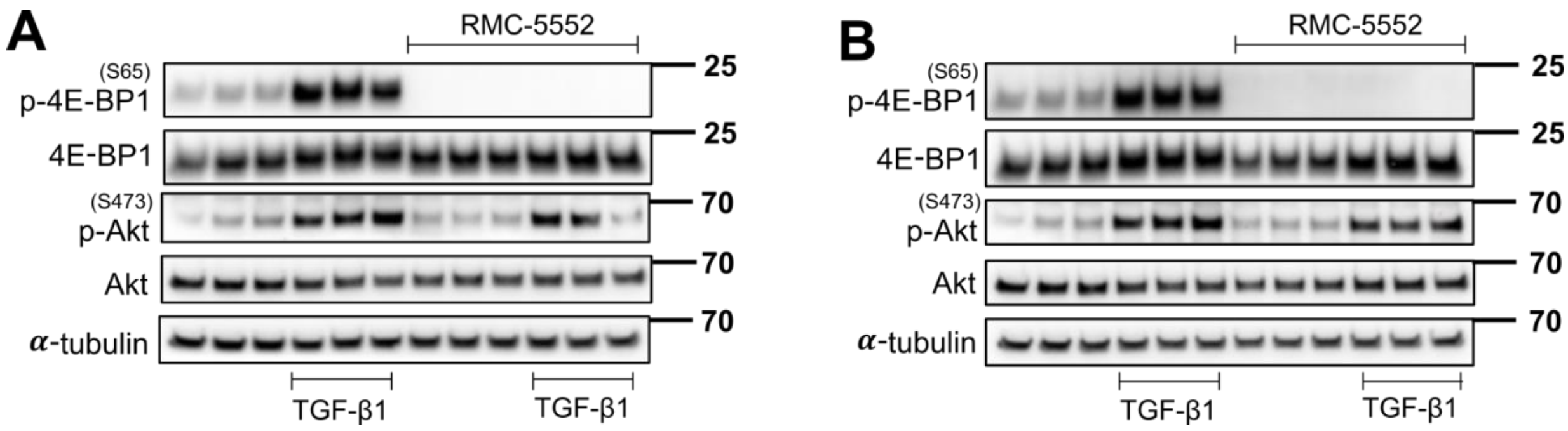

The mTORC1-specific inhibitor RMC-5552 inhibits TGF-β1-induced mTORC1 signaling but not mTORC2 signaling in pHLFs. (A, B) Independent repeats of the impact of RMC-5552 on the phosphorylation of specified proteins shown by Western blot for mTORC1 and mTORC2 signalling to complement Figure 5E for two additional passages of the same donor line. Protein band size is shown in KDa. Following electrophoresis, proteins on the gel were transferred to nitrocellulose membranes as described in the Materials and Methods section. The membrane was cut in two. The bottom section was first probed for the phosphorylated forms of 4E-BP1, then stripped with Restore™ Western Blot Stripping Buffer (ThermoFisher) and probed for total protein levels of 4E-BP1. This was repeated for p-AKT and AKT on the top section. Finally, the top section was stripped a second time and probed for α-tubulin as the loading control. Nitrocellulose membranes probed for each protein are shown in Figure S10.

Fig. S10.

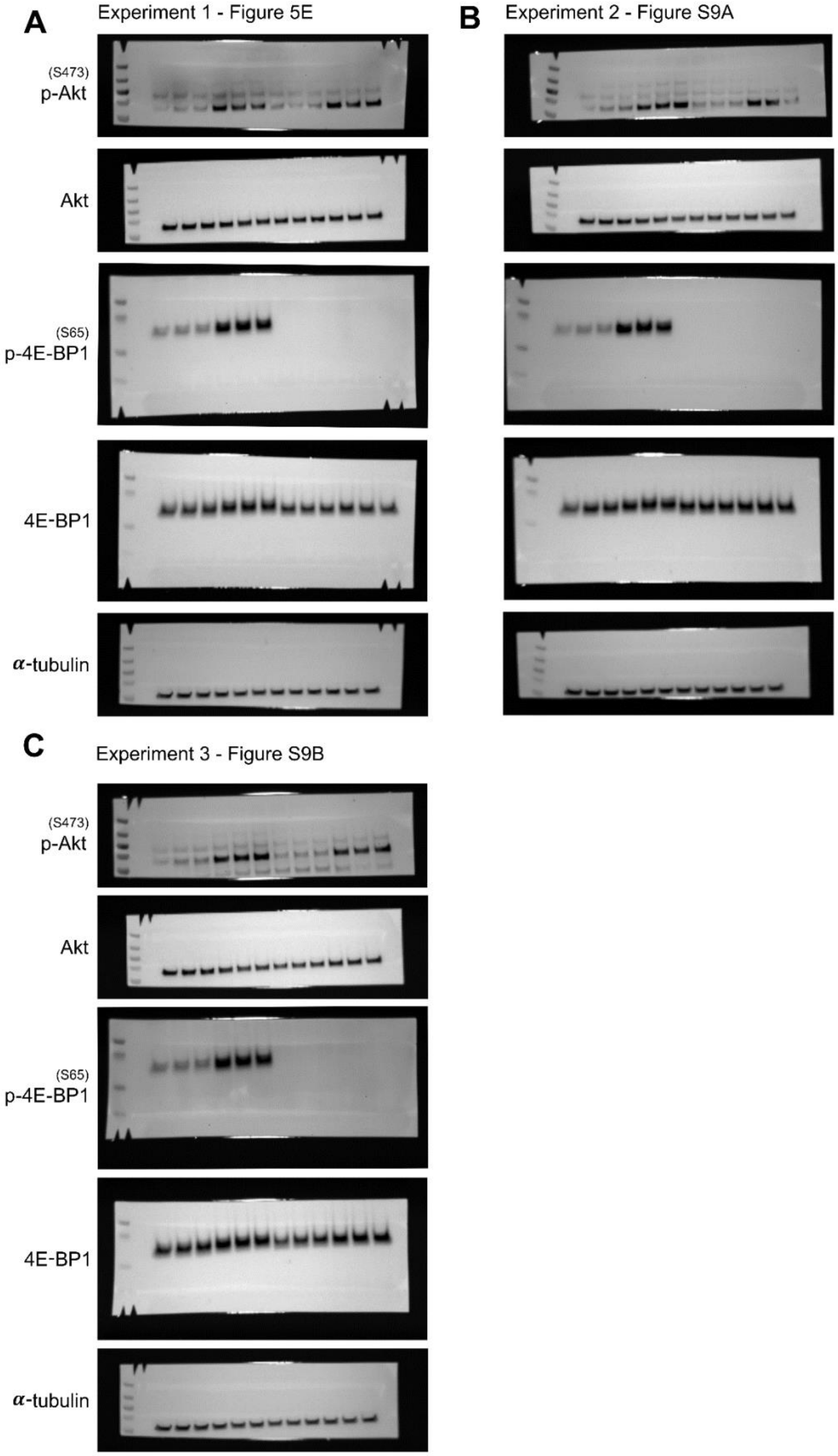
A
Experiment 1 - Figure 5E
(S473)
p-Akt
Akt
(S65)
p-4E-BP1
4E-BP1
α-tubulin
B
Experiment 2 - Figure S9A
C
Experiment 3 - Figure S9B
(S473)
p-Akt
Akt
(S65)
p-4E-BP1
4E-BP1
α-tubulin

Images of original membranes for Western Blots in Figures 5E and S9A and B. The figure shows the images of the membranes probed for the indicated proteins corresponding to Figure 5E (A) and Supplementary Figure S9A (B) and B (C). Following electrophoresis, proteins on the gel were transferred to nitrocellulose membranes as described in the Materials and Methods section. The membrane was cut in two. The bottom section was first probed for the phosphorylated forms of 4E-BP1, then stripped with Restore™ Western Blot Stripping Buffer (ThermoFisher) and probed for total protein levels of 4E-BP1. This was repeated for p-AKT and AKT on the top section. Finally, the top section was stripped a second time and probed for α-tubulin as the loading control.

Fig. S11.

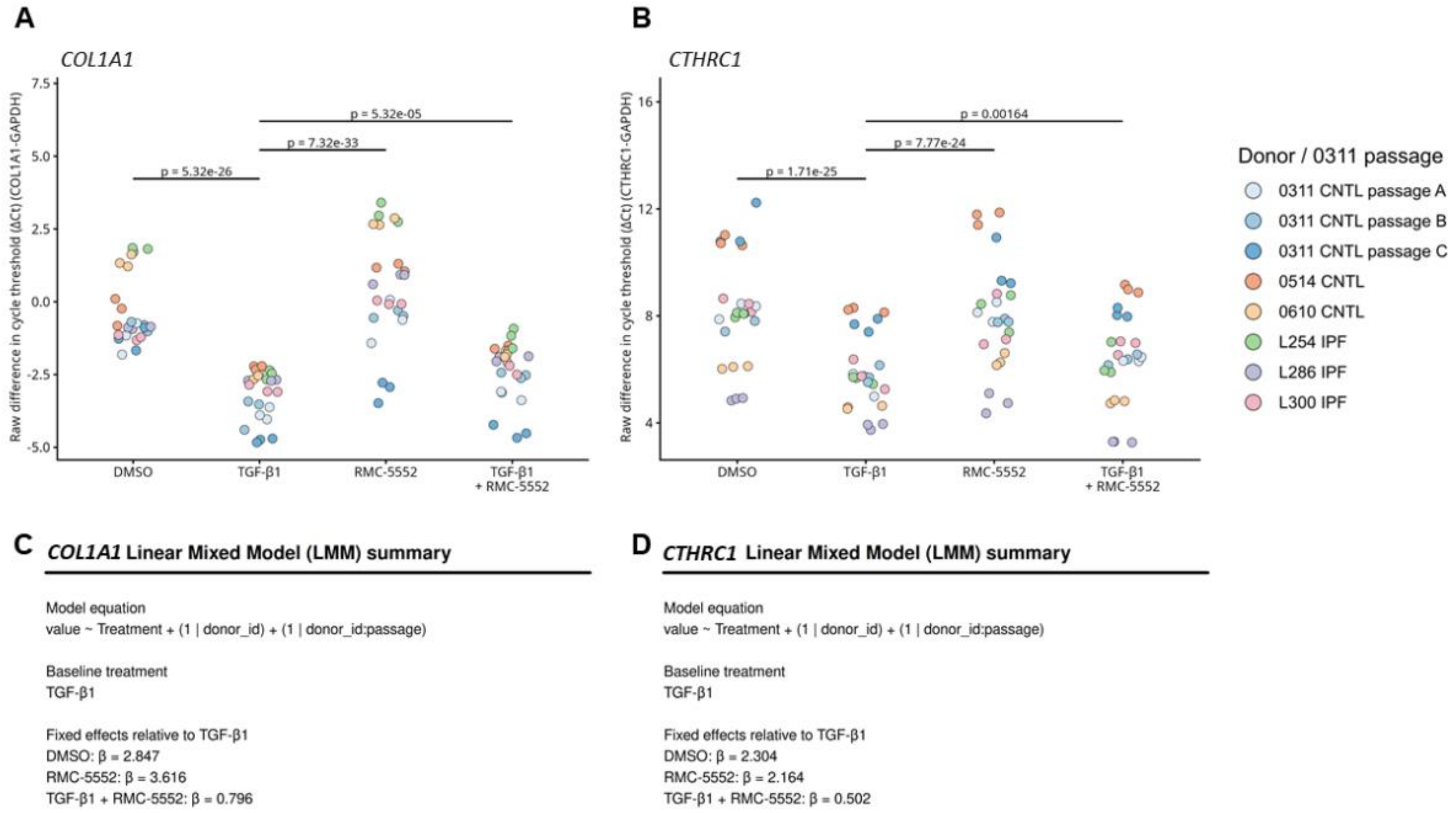

Linear Mixed-Effects Model (LMM) raw data and equations summary. A and B: Plots of delta cycle threshold values (ΔCt) showing all technical repeats across all passages and biological donor origin of cell lines for *COL1A1* and *CTHRC1* qPCR data. **C and D:** Equations of fitted linear mixed-effects models.

REFERENCES

1. N. C. Henderson, F. Rieder, & T. A. Wynn, (2020). Fibrosis:from mechanisms to medicines. Nature, 587(7835), 555-566. https://doi.org/10.1038/s41586-020-2938-9.

2. J. Kaunisto, E.-R. Salomaa, U. Hodgson, R. Kaarteenaho, H. Kankaanranta, K. Koli, T. Vahlberg, M. Myllärniemi, Demographics and survival of patients with idiopathic pulmonary fibrosis in the Finnish IPF registry. *ERJ Open Res.* **5**, 00170–02018 (2019).

3. C. C. Moor, R. L. M. Mostard, J. C. Grutters, P. Bresser, J. G. J. V. Aerts, C. D. Dirksen, M. L. Kimman, M. S. Wijsenbeek, Patient expectations, experiences and satisfaction with nintedanib and pirfenidone in idiopathic pulmonary fibrosis: A quantitative study. *Respir. Res.* **21**, 196 (2020).

4. A. J. Podolanczuk, C. C. Thomson, M. Remy-Jardin, L. Richeldi, F. J. Martinez, M. Kolb, G. Raghu, Idiopathic pulmonary fibrosis: State of the art for 2023. *Eur. Respir. J.* **61**, 2200957 (2023).

5. B. Selvarajah, M. Platé, R. C. Chambers, Pulmonary fibrosis: Emerging diagnostic and therapeutic strategies. *Mol. Aspects Med.* **94**, 101227 (2023).

6. K. Tsoyi, I. O. Rosas, Fibroblast heterogeneity in pulmonary fibrosis: A new target for therapeutics development? *Eur. Respir. J.* **63**, 2302188 (2024).

7. A. C. Habermann, A. J. Gutierrez, L. T. Bui, S. L. Yahn, N. I. Winters, C. L. Calvi, L. Peter, M.-I. Chung, C. J. Taylor, C. Jetter, L. Raju, J. Roberson, G. Ding, L. Wood, J. M. S. Sucre, B. W. Richmond, A. P. Serezani, W. J. McDonnell, S. B. Mallal, M. J. Bacchetta, J. E. Loyd, C. M. Shaver, L. B. Ware, R. Bremner, R. Walia, T. S. Blackwell, N. E. Banovich, J. A. Kropski, Single-cell RNA sequencing reveals profibrotic roles of distinct epithelial and mesenchymal lineages in pulmonary fibrosis. *Sci. Adv.* **6**, (2020).

8. T. Tsukui, K.-H. Sun, J. B. Wetter, J. R. Wilson-Kanamori, L. A. Hazelwood, N. C. Henderson, T. S. Adams, J. C. Schupp, S. D. Poli, I. O. Rosas, N. Kaminski, M. A. Matthay, P. J. Wolters, D. Sheppard, Collagen-producing lung cell atlas identifies multiple subsets with distinct localization and relevance to fibrosis. *Nat. Commun.* **11**, 1920 (2020).

9. A. Ruiz-Villalba, J. P. Romero, S. C. Hernández, A. Vilas-Zornoza, N. Fortelny, L. Castro-Labrador, P. San Martin-Uriz, E. Lorenzo-Vivas, P. García-Olloqui, M. Palacio, J. J. Gavira, G. Bastarrika, S. Janssens, M. Wu, E. Iglesias, G. Abizanda, X. M. de Morentin, M. Lasaga, N. Planell, C. Bock, D. Alignani, G. Medal, I. Prudovsky, Y.-R. Jin, S. Ryzhov, H. Yin, B. Pelacho, D. Gomez-Cabrero, V. Lindner, D. Lara-Astiaso, F. Prósper, Single-Cell RNA Sequencing Analysis Reveals a Crucial Role for CTHRC1 (Collagen Triple Helix Repeat Containing 1) Cardiac Fibroblasts After Myocardial Infarction. *Circulation* **142**, 1831–1847 (2020).

10. X.-L. Zhang, L.-P. Hu, Q. Yang, W.-T. Qin, X. Wang, C.-J. Xu, G.-A. Tian, X.-M. Yang, L.-L. Yao, L. Zhu, H.-Z. Nie, Q. Li, Q. Xu, Z.-G. Zhang, Y.-L. Zhang, J. Li, Y.-H. Wang, S.-H. Jiang, CTHRC1 promotes liver metastasis by reshaping infiltrated macrophages through physical interactions with TGF-β receptors in colorectal cancer. *Oncogene* **40**, 3959–3973 (2021).

11. S. L. Friedman, D. Sheppard, J. S. Duffield, S. Violette, Therapy for fibrotic diseases: Nearing the starting line. *Sci. Transl. Med.* **5**, 167sr1 (2013).

12. W. Z. Mehal, J. Iredale, S. L. Friedman, Scraping fibrosis: Expressway to the core of fibrosis. *Nat. Med.* **17**, 552–553 (2011).

13. N. C. Henderson, T. D. Arnold, Y. Katamura, M. M. Giacomini, J. D. Rodriguez, J. H. McCarty, A. Pellicoro, E. Raschperger, C. Betsholtz, P. G. Ruminski, D. W. Griggs, M. J. Prinsen, J. J. Maher, J. P. Iredale, A. Lacy-Hulbert, R. H. Adams, D. Sheppard, Targeting of αv integrin identifies a core molecular pathway that regulates fibrosis in several organs. *Nat. Med.* **19**, 1617–1624 (2013).

14. F. Verrecchia, M.-L. Chu, A. Mauviel, Identification of Novel TGF-β/Smad gene targets in dermal fibroblasts using a combined cdna microarray/promoter transactivation approach. *J. Biol. Chem.* **276**, 17058–17062 (2001).

15. Z. Mukhatayev, A. Adilbayeva, J. Kunz, CTHRC1: An emerging hallmark of pathogenic fibroblasts in lung fibrosis. *Cells* **13**, 946 (2024).

16. H. V. Woodcock, J. D. Eley, D. Guillotin, M. Platé, C. B. Nanthakumar, M. Martufi, S. Peace, G. Joberty, D. Poeckel, R. B. Good, A. R. Taylor, N. Zinn, M. Redding, E. J. Forty, R. E. Hynds, C. Swanton, M. Karsdal, T. M. Maher, G. Bergamini, R. P. Marshall, A. D. Blanchard, P. F. Mercer, R. C. Chambers, The mTORC1/4E-BP1 axis represents a critical signaling node during fibrogenesis. *Nat. Commun.* **10**, 6 (2019).

17. J. Huang, B. D. Manning, A complex interplay between Akt, TSC2 and the two mTOR complexes. *Biochem. Soc. Trans.* **37**, 217–222 (2009).

18. H. Hua, Q. Kong, H. Zhang, J. Wang, T. Luo, Y. Jiang, Targeting mTOR for cancer therapy. *J. Hematol. Oncol.* **12**, 71 (2019).

19. D. Meng, X. Zhao, Y. C. Yang, A. Navickas, C. Helland, H. Goodarzi, M. Singh, S. Bandyopadhyay, A bi-steric mTORC1-selective inhibitor overcomes drug resistance in breast cancer. *Oncogene* **42**, 2207–2217 (2023).

20. A. M. Schram, A. R. Naqash, E. B. Haura, J. W. Riess, S. V. Ulahannan, S.-H. I. Ou, A. Capasso, P. N. Munster, M. L. Cheng, W. C. Gustafson, B. Bitman, S. Kar, Z. Wang, L. Tao, J. G. Meyerowitz, H. A. Burris, Abstract C020: First-in-human phase 1/1b trial of the first-in-class bi-steric mTORC1-selective inhibitor RMC-5552 in patients with advanced solid tumors. *Mol. Cancer Ther.* **22**, C020–C020 (2023).

21. B. Selvarajah, I. Azuelos, M. Platé, D. Guillotin, E. J. Forty, G. Contento, H. V. Woodcock, M. Redding, A. Taylor, G. Brunori, P. F. Durrenberger, R. Ronzoni, A. D. Blanchard, P. F. Mercer, D. Anastasiou, R. C. Chambers, mTORC1 amplifies the ATF4-dependent de novo serine-glycine pathway to supply glycine during TGF-β_1—induced collagen biosynthesis. *Sci. Signal.* **12**, eaav3048 (2019).

22. A. Biernacka, M. Dobaczewski, N. G. Frangogiannis, TGF-β signaling in fibrosis. *Growth Factors* **29**, 196–202 (2011).

23. A. B. Roberts, Is Smad3 a major player in signal transduction pathways leading to fibrogenesis? *Chest* **120**, 43S–47S (2001).

24. T. S. Adams, J. C. Schupp, S. Poli, E. A. Ayaub, N. Neumark, F. Ahangari, S. G. Chu, B. A. Raby, G. DeIuliis, M. Januszyk, Q. Duan, H. A. Arnett, A. Siddiqui, G. R. Washko, R. Homer, X. Yan, I. O. Rosas, N. Kaminski, Single-cell RNA-seq reveals ectopic and aberrant lung-resident cell populations in idiopathic pulmonary fibrosis. *Sci. Adv.* **6**, eaba1983 (2020).

25. T. Tsukui, P. J. Wolters, D. Sheppard, Alveolar fibroblast lineage orchestrates lung inflammation and fibrosis. *Nature* **631**, 627–634 (2024).

26. D. Sun, X. Guan, A. E. Moran, L.-Y. Wu, D. Z. Qian, P. Schedin, M.-S. Dai, A. V. Danilov, J. J. Alumkal, A. C. Adey, P. T. Spellman, Z. Xia, Identifying phenotype-associated subpopulations by integrating bulk and single-cell sequencing data. *Nat. Biotechnol.* **40**, 527–538 (2022).

27. X. Sun, A.-K. Perl, R. Li, S. M. Bell, E. Sajti, V. V. Kalinichenko, T. V. Kalin, R. S. Misra, H. Deshmukh, G. Clair, J. Kyle, L. E. C. Alexander, J. A. Masso-Silva, J. A. Kitzmiller, K. A. Wikenheiser-Brokamp, G. Deutsch, M. Guo, Y. Du, M. P. Morley, M. J. Valdez, H. V. Yu, K. Jin, E. E. Bardes, J. A. Zepp, T. Neithamer, M. C. Basil, W. J. Zacharias, J. Verheyden, R. Young, G. Bandyopadhyay, S. Lin, C. Ansong, J. Adkins, N. Salomonis, B. J. Aronow, Y. Xu, G. Pryhuber, J. Whitsett, E. E. Morrisey, A census of the lung: CellCards from LungMAP. *Dev. Cell* **57**, 112–145.e2 (2022).

28. M. Corselli, C.-W. Chen, B. Sun, S. Yap, J. P. Rubin, B. Péault, The tunica adventitia of human arteries and veins as a source of mesenchymal stem cells. *Stem Cells Dev.* **21**, 1299–1308 (2012).

29. X. Zhang, X. Shi, F. Xie, Y. Liu, X. Wei, Y. Cai, J. Chao, Dissecting pulmonary fibroblasts heterogeneity in lung development, health and diseases. *Heliyon* **9**, e19428 (2023).

30. C. H. Mayr, A. Sengupta, S. Asgharpour, M. Ansari, J. C. Pestoni, P. Ogar, I. Angelidis, A. Liontos, J. A. Rodriguez-Castillo, N. J. Lang, M. Strunz, D. Porras-Gonzalez, M. Gerckens, L. J. De Sadeleer, B. Oehrle, V. Viteri-Alvarez, I. E. Fernandez, M. Tallquist, M. Irmler, J. Beckers, O. Eickelberg, G. M. Stoleriu, J. Behr, N. Kneidinger, W. A. Wuyts, R. M. Wasnick, A. Ö. Yildirim, K. Ahlbrecht, R. E. Morty, C. Samakovlis, F. J. Theis, G. Burgstaller, H. B. Schiller, Sfrp1 inhibits lung fibroblast invasion during transition to injury-induced myofibroblasts. *Eur. Respir. J.* **63**, 2301326 (2024).

31. J.-P. Ng-Blichfeldt, T. de Jong, R. K. Kortekaas, X. Wu, M. Lindner, V. Guryev, P. S. Hiemstra, J. Stolk, M. Königshoff, R. Gosens, TGF-β activation impairs fibroblast ability to support adult lung epithelial progenitor cell organoid formation. *Am. J. Physiol. Lung Cell. Mol. Physiol.* **317**, L14–L28 (2019).

32. F. Klingberg, B. Hinz, E. S. White, The myofibroblast matrix: Implications for tissue repair and fibrosis. *J. Pathol.* **229**, 298–309 (2013).

33. G. Serini, M.-L. Bochaton-Piallat, P. Ropraz, A. Geinoz, L. Borsi, L. Zardi, G. Gabbiani, The fibronectin domain ED-A is crucial for myofibroblastic phenotype induction by transforming growth factor-β1. *J. Cell Biol.* **142**, 873–881 (1998).

34. M. Kohan, A. F. Muro, E. S. White, N. Berkman, EDA-containing cellular fibronectin induces fibroblast differentiation through binding to α 4 β 7 integrin receptor and MAPK/Erk 1/2-dependent signaling. *FASEB J.* **24**, 4503–4512 (2010).

35. J. Li, J. Cao, M. Li, Y. Yu, Y. Yang, X. Xiao, Z. Wu, L. Wang, Y. Tu, H. Chen, Collagen triple helix repeat containing-1 inhibits transforming growth factor-β1-induced collagen type I expression in keloid. *Br. J. Dermatol.* **164**, 1030–1036 (2011).

36. Z. Shen, T. Su, J. Chen, Z. Xie, J. Li, Collagen triple helix repeat containing-1 exerts antifibrotic effects on human skin fibroblast and bleomycin-induced dermal fibrosis models. *Ann. Transl. Med.* **9**, 801–801 (2021).

37. P. Pyagay, M. Heroult, Q. Wang, W. Lehnert, J. Belden, L. Liaw, R. E. Friesel, V. Lindner, Collagen triple helix repeat containing 1, a novel secreted protein in injured and diseased arteries, inhibits collagen expression and promotes cell migration. *Circ. Res.* **96**, 261–268 (2005).

38. M. Cao, D. Ke, H. Zhou, The role and molecular mechanism of CTHRC1 in fibrosis. *Life Sci.* **350**, 122745 (2024).

39. A. P. Binks, M. Beyer, R. Miller, R. J. LeClair, Cthrc1 lowers pulmonary collagen associated with bleomycin-induced fibrosis and protects lung function. *Physiol. Rep.* **5**, e13115 (2017).

40. K. Yang, N. Huang, J. Sun, W. Dai, M. Chen, J. Zeng, Transforming growth factor-β induced protein regulates pulmonary fibrosis via the G-protein signaling modulator 2 /Snail axis. *Peptides* **155**, 170842 (2022).

41. D. Guillotin, A. R. Taylor, M. Platé, P. F. Mercer, L. M. Edwards, R. Haggart, G. Miele, R. J. Mcanulty, T. M. Maher, R. E. Hynds, M. Jamal-Hanjani, R. P. Marshall, A. J. Fisher, A. D. Blanchard, R. C. Chambers, Transcriptome analysis of IPF fibroblastic foci identifies key pathways involved in fibrogenesis. *Thorax* **76**, 73–82 (2021).

42. P. K. Naik, P. D. Bozyk, J. K. Bentley, A. P. Popova, C. M. Birch, C. A. Wilke, C. D. Fry, E. S. White, T. H. Sisson, N. Tayob, B. Carnemolla, P. Orecchia, K. R. Flaherty, M. B. Hershenson, S. Murray, F. J. Martinez, B. B. Moore, Periostin promotes fibrosis and predicts progression in patients with idiopathic pulmonary fibrosis. *Am. J. Physiol. Lung Cell. Mol. Physiol.* **303**, L1046–L1056 (2012).

43. Y. Tian, H. Li, Y. Gao, C. Liu, T. Qiu, H. Wu, M. Cao, Y. Zhang, H. Ding, J. Chen, H. Cai, Quantitative proteomic characterization of lung tissue in idiopathic pulmonary fibrosis. *Clin. Proteomics* **16**, 6 (2019).

44. S. Huang, X. Lai, L. Yang, F. Ye, C. Huang, Y. Qiu, S. Lin, L. Pu, Z. Wang, W. Huang, Asporin promotes TGF-β–induced lung myofibroblast differentiation by facilitating Rab11-dependent recycling of TβRI. *Am. J. Respir. Cell Mol. Biol.* **66**, 158–170 (2022).

45. S. Dulauroy, S. E. Di Carlo, F. Langa, G. Eberl, L. Peduto, Lineage tracing and genetic ablation of $ADAM12^+$ perivascular cells identify a major source of profibrotic cells during acute tissue injury. *Nat. Med.* **18**, 1262–1270 (2012).

46. R. J. Allen, B. Guillen-Guio, J. M. Oldham, S. F. Ma, A. Dressen, M. L. Paynton, L. M. Kraven, M. Obeidat, X. Li, M. Ng, R. Braybrooke, M. Molina-Molina, B. D. Hobbs, R. K. Putman, P. Sakornsakolpat, H. L. Booth, W. A. Fahy, S. P. Hart, M. R. Hill, N. Hirani, R. B. Hubbard, R. J. McAnulty, A. B. Millar, V. Navaratnam, E. Oballa, H. Parfrey, G. Saini, M. K. B. Whyte, Y. Zhang, N. Kaminski, A. Adegunsoye, M. E. Strek, M. Neighbors, X. R. Sheng, G. Gudmundsson, V. Gudnason, H. Hatabu, D. J. Lederer, A. Manichaikul, J. D. Newell, G. T. O'Connor, V. E. Ortega, H. Xu, T. E. Fingerlin, Y. Bossé, K. Hao, P. Joubert, D. C. Nickle,

D. D. Sin, W. Timens, D. Furniss, A. P. Morris, K. T. Zondervan, I. P. Hall, I. Sayers, M. D. Tobin, M. Toby, M. H. Cho, G. M. Hunninghake, D. A. Schwartz, B. L. Yaspan, P. L. Molyneaux, C. Flores, I. Noth, R. G. Jenkins, L. V. Wain, Genome-wide association study of susceptibility to idiopathic pulmonary fibrosis. *Am. J. Respir. Crit. Care Med.* **201**, 564–574 (2020).

47. The Galaxy Community, The Galaxy platform for accessible, reproducible and collaborative biomedical analyses: 2022 update. *Nucleic Acids Res.* **50**, W345–W351 (2022).

48. P. Ewels, M. Magnusson, S. Lundin, M. Käller, MultiQC: Summarize analysis results for multiple tools and samples in a single report. *Bioinformatics* **32**, 3047–3048 (2016).

49. S. Andrews, FastQC: A Quality Control Tool for High Throughput Sequence Data. Babraham Bioinformatics, Babraham Institute (2016); https://www.bioinformatics.babraham.ac.uk/projects/fastqc/.

50. D. Kim, B. Langmead, S. L. Salzberg, HISAT: a fast spliced aligner with low memory requirements. *Nat. Methods* **12**, 357–360 (2015).

51. Y. Liao, G. K. Smyth, W. Shi, featureCounts: An efficient general purpose program for assigning sequence reads to genomic features. *Bioinformatics* **30**, 923–930 (2014).

52. M. I. Love, W. Huber, S. Anders, Moderated estimation of fold change and dispersion for RNA-seq data with DESeq2. *Genome Biol.* **15**, 550 (2014).

53. R Core Team, R: A Language and Environment for Statistical Computing. R Foundation for Statistical Computing, Vienna, Austria (2016); www.R-project.org.

54. RStudio Team, RStudio: Integrated Development for R (RStudio, 2020); www.rstudio.com/.

55. T. Wu, E. Hu, S. Xu, M. Chen, P. Guo, Z. Dai, T. Feng, L. Zhou, W. Tang, L. Zhan, X. Fu, S. Liu, X. Bo, G. Yu, clusterProfiler 4.0: A universal enrichment tool for interpreting omics data. *Innovation* **2**, 100141 (2021).

56. G. Yu, L.-G. Wang, Y. Han, Q.-Y. He, clusterProfiler: An R Package for comparing biological themes among gene clusters. *OMICS* **16**, 284–287 (2012).

57. K. Blighe, S. Rana, M. Lewis, EnhancedVolcano: Publication-ready volcano plots with enhanced colouring and labeling. R package version 1.01, Bioconductor (2019); https://bioconductor.org/packages/EnhancedVolcano.

58. R. Kolde, Pheatmap: pretty heatmaps. R package version 0.7.7, CRAN (2012); https://CRAN.R-project.org/package=pheatmap58.

59. Y. Hao, T. Stuart, M. H. Kowalski, S. Choudhary, P. Hoffman, A. Hartman, A. Srivastava, G. Molla, S. Madad, C. Fernandez-Granda, R. Satija, Dictionary learning for integrative, multimodal and scalable single-cell analysis. *Nat. Biotechnol.* **42**, 293–304 (2024).
