# Supplementary material for: Critical role for the TGF-β1/mTORC1 signaling axis in defining the transcriptional identity of CTHRC1+ pathologic fibroblasts
Source: Sci Adv. 2026 Sep 2;12(36):eadx9868. doi: 10.1126/sciadv.adx9868 (PMC13537237; doi:10.1126/sciadv.adx9868)
Supplement: Supplementary file 2 [file sciadv.adx9868_tables_s4_to_s10.zip › adx9868_tables_s4_to_s10.html]

Supplementary Tables


# Supplementary Tables

#### 2025-10-18

### Contents

This document contains the supplementary data tables for the
transcriptomic analyses described:

- Tables S4-S6 - Pharmacological datasets - RNA-Seq data from pHLFs
  treated with AZD8055 or rapamycin
- Tables S7-S8 - CRISPR/Cas9 datasets - RNA-Seq data from CRISPR/Cas9
  gene edited pHLFs
- Tables S9-S10 - Scissor analysis - mapping RNA-Seq data onto a human
  IPF scRNA-Seq dataset (GSE136831)

### Table S4

### Table S5

### Table S6

### Table S7

### Table S8

### Table S9

### Table S10
